# Crosstalk Between Signaling Pathways Involved in the Regulation of Airway Smooth Muscle Cell Hyperplasia

**DOI:** 10.3389/fphar.2019.01148

**Published:** 2019-10-09

**Authors:** Hui Min Yap, Daud Ahmad Israf, Hanis Hazeera Harith, Chau Ling Tham, Mohd Roslan Sulaiman

**Affiliations:** Department of Biomedical Science, Faculty of Medicine and Health Sciences, Universiti Putra Malaysia, Serdang, Malaysia

**Keywords:** airway remodeling, ASM hyperplasia, ASM cell proliferation, ASM cell migration, ASM cell apoptosis

## Abstract

Increased ASM mass, primarily due to ASM hyperplasia, has been recognized as a hallmark of airway remodeling in asthma. Increased ASM mass is the major contributor to the airway narrowing, thus worsening the bronchoconstriction in response to stimuli. Inflammatory mediators and growth factors released during inflammation induce increased ASM mass surrounding airway wall *via* increased ASM proliferation, diminished ASM apoptosis and increased ASM migration. Several major pathways, such as MAPKs, PI3K/AKT, JAK2/STAT3 and Rho kinase, have been reported to regulate these cellular activities in ASM and were reported to be interrelated at certain points. This article aims to provide an overview of the signaling pathways/molecules involved in ASM hyperplasia as well as the mapping of the interplay/crosstalk between these major pathways in mediating ASM hyperplasia. A more comprehensive understanding of the complexity of cellular signaling in ASM cells will enable more specific and safer drug development in the control of asthma.

## Introduction

Airway inflammation and airway hyperresponsiveness (AHR) are both indispensable features of asthma (Busse, 2010). AHR can be defined as an exaggerated response of the airways to a variety of stimuli, such as histamine, methacholine, or cold air that result in airway obstruction (Postma and Kerstjens, 1998; Meurs et al., 2008). Persistent allergen challenge that correlates with chronic airway inflammation sustained by mediators released by infiltrating as well as resident cells, will ultimately lead to airway remodeling (Lloyd and Robinson, 2007; Khan, 2013; Xia et al., 2013). Airway remodeling, a collective term for the structural changes of the asthmatic airway, can be characterized by increased airway smooth muscle (ASM) mass, goblet cell hyperplasia and subepithelial fibrosis (Fehrenbach et al., 2017). The ASM has been identified as the principal regulator of AHR in asthma due to its increased contractibility and mass in response to inflammatory mediators (Hershenson et al., 2008; Martin and Lauzon, 2016). Studies have demonstrated that the thickened layer of ASM causes airway narrowing (structural changes) as well as exaggerated ASM shortening (contraction) in response to contractile agonists (Ijpma et al., 2017; Oliver et al., 2007). These findings suggest that increased ASM mass contributes both directly and indirectly to AHR, hence associated with the severity of asthma. The clinical usage of bronchial thermoplasty (BT) therapy reflects the importance of ASM hyperplasia in severe asthma. BT is a Food and Drug Administration (FDA) approved endoscopic therapy for severe and persistent asthma (refractory asthma) (Menzella et al., 2017) that delivers radio frequency thermal energy to the airways through catheterization of the proximal bronchus (Dombret et al., 2014). BT is applied mainly to reduce ASM mass, thus reducing the bronchospasms and AHR (Pretolani et al., 2014). Furthermore, there is evidence demonstrating that BT is also capable of reducing airway inflammation (Denner et al., 2015). There have been controversial views regarding the application of BT which include lack of understanding of the underlying mechanism of action coupled with fact that some asthmatics are unresponsive to the treatment (Doeing et al., 2013; Dombret et al., 2014; Menzella et al., 2017). Doeing et al. (2013) demonstrated that BT failed to reduce ASM hyperplasia in a patient with severe persistent asthma. This study highlighted the need to increase the temperature applied for BT to reduce ASM and the need to further study the effect of BT on ASM ablation especially in severe asthmatic patients. Nevertheless, BT still remains as an approved therapeutic option for refractory asthma that is non-responsive towards standard asthma treatment.

## Effects of Current Asthma Medicines

Current asthma therapies act mainly by attenuating ASM contraction and suppressing airway inflammation with minimal effects upon prevention and/or reversal of ASM remodeling (Keglowich and Borger, 2015). Combination therapy with inhaled corticosteroids (ICS) and long-acting β agonists (LABAs) remains the mainstay pharmacotherapeutic approach in asthma management (Ye et al., 2017). ICS are effective in suppressing inflammation while LABAs act as bronchodilators (Charriot et al., 2016). Airway remodeling is thought to be a secondary event in asthma following persistent airway inflammation. However, Baraldo et al. (2011) demonstrated that the airway remodeling occurs in asthmatic children in the absence of eosinophilic inflammation. Furthermore, young children with chronic asthma have detectable markers of airway remodeling and inflammation (Malmstrom et al., 2013). These findings suggest that airway remodeling may occur in parallel with airway inflammation. Therefore, therapy that can reverse or inhibit both airway inflammation and airway remodeling would be of great benefit in the management of asthma. The therapeutic effect of combined ICS and LABA treatment in reversing ASM remodeling remains controversial. In an equine asthma model, combination treatment with corticosteroids and LABAs and corticosteroids alone were demonstrated to decrease ASM hyperplasia (Bullone et al., 2017). *In vitro* studies with human cells demonstrated that combined corticosteroid and LABA treatment was ineffective in preventing or reversing remodeling events such as extracellular matrix (ECM) deposition and the release of interleukin-6 (IL-6) in both asthmatic and non-asthmatic ASM cells (Ge et al., 2012), whereas omalizumab, an anti-IgE mAb, when used as an add-on treatment to corticosteroids and LABA, was shown to reduce both airway inflammation and airway remodeling (Hoshino and Ohtawa, 2012). Alternatively, treatment with the corticosteroid dexamethasone caused mix responses in terms of its inhibitory effect upon mitogen-induced ASM proliferation in human non-asthmatic ASM cells (Fernandes et al., 1999; Bonacci et al., 2003; Panettieri, 2004). An in-depth understanding of the underlying mechanism of increased ASM mass is therefore crucial for the development of therapeutic strategies that directly target altered ASM physiology leading to more effective management of asthma.

Other less commonly used treatment modalities in the management of asthma include leukotriene receptor antagonists (LTRAs), anticholinergics and monoclonal antibody (mAb) therapies appear. The action of LTRAs results in both bronchodilator and anti-inflammatory effects whereby anticholinergics, particularly long-acting muscarinic antagonists (LAMAs), work as bronchodilators (Dempsey, 2000; Maria et al., 2017). Treatment with a combination of oral LTRA, such as montelukast with ICS and LABA was shown to improve airway function, but not airway remodeling in moderate-to-severe asthma patients (Gao et al., 2013), whereas tiotropium bromide, a LAMA, has been demonstrated to inhibit ASM remodeling in a guinea pig model of allergic asthma (Gosens et al., 2005). The first mAb therapy approved for asthma treatment was omalizumab which is effective in neutralizing the IgE-mediated allergic cascade in asthma (D’Amato et al., 2014; Maria et al., 2017). IgE has been suggested to also induce proliferation and secretion of proinflammatory cytokines in human non-asthmatic ASM cells and omalizumab has been reported to significantly attenuate these effects (Roth and Tamm, 2010; Redhu et al., 2013; Roth et al., 2013).

The role of T cells in the pathophysiology of asthma is well documented. The role of the T helper 17 (Th17) cell and its cytokine in airway remodeling has been reported and reviewed in recent years (Pain et al., 2014; Gu et al., 2017; Camargo et al., 2018). The Th17 cytokine IL-17 has been shown to induce bronchial epithelial cells to produce insulin-like growth factor-І (IGF-І), which is known to induce collagen formation as well as ASM hyperplasia (Goldstein et al., 1989; Noveral et al., 1994; Kawaguchi et al., 2010). Furthermore, a previous study has demonstrated that IL-17 acts upon human bronchial fibroblasts to produce cytokines, such as growth-related oncogene alpha (Gro-α)/CXCL1, which was reported to inhibit human airway smooth muscle cell migration (Molet et al., 2001; Al-Alwan et al., 2014; Pain et al., 2014). In addition, the anti-IL-17 mAb has been shown to reduce the levels of several remodeling markers, which include TGF-β, fibronectin, collagen fibers І and MMP-9, in a murine asthma model (Camargo et al., 2018). Th17-associated cytokines have been demonstrated to induce ASM cell proliferation, migration, and reduced ASM cell apoptosis (Chang et al., 2011; Chang et al., 2012), suggesting that Th17-associated cytokines possibly contribute to ASM hyperplasia in asthma. T helper 2 (Th2) cells on the other hand have been recognized for their role in mediating IgE synthesis through production of interleukin (IL)-4 and IL-13 (Romagnani, 2004). Inhibition of the Th2 cytokine IL-13 with an anti-IL-13 mAb has been shown to inhibit airway remodeling in a chronic mouse model of asthma (Blease et al., 2001; Yang et al., 2004). Furthermore, Th2 cytokines were shown to enhance ASM proliferation and migration leading to ASM remodeling (Parameswaran et al., 2007; Moynihan et al., 2008). Since both Th2 and Th17 cells play a significant role in ASM hyperplasia, mAb therapy targeting both Th2 and Th17-associated cytokines has good potential in reducing ASM remodeling in asthma.

## Mechanism of Increased ASM Mass

Increased ASM mass can result from hyperplasia and/or hypertrophy. ASM hypertrophy is reported to be present in both fatal and non-fatal asthma while ASM hyperplasia is predominantly detected in cases of fatal asthma (James et al., 2012). This suggests that an increase in ASM cell number, possibly due to proliferative responses, is more likely to contribute to the severity of asthma. Increased ASM mass has been linked to increased ASM proliferation and migration and diminished ASM apoptosis (Ozier et al., 2011; Berair et al., 2013). Higher expression of cellular proliferation markers (PCNA and/or Ki-67) coincide with increased ASM cell proliferation rates in asthmatic patients (Woodruff et al., 2004; Hassan et al., 2010; Ramos-Barbon et al., 2010). Alternatively, opposing arguments claim no differences in the number of PCNA and/or Ki-67 positive ASM cells between human asthmatic and non-asthmatic airways (Ijpma et al., 2017; Naveed et al., 2017). Ijpma et al. (2017) claim that discrepancies could stem from the variation that occurs during data collection. In addition, the findings reported by Ijpma et al. (2017) have demonstrated that there was no correlation between PCNA- and cyclin D-positive cells. Besides, PCNA has been reported to be upregulated for its role in DNA repair and chromatin remodeling, implying the expression of PCNA could increase due to DNA repair or chromatin remodeling without any increment in cell number (Maga and Hubscher, 2003). Therefore, the sensitivity of these molecules (Ki-67, PCNA and cyclin D) as proliferation markers has been questioned by Ijpma et al. (2017). Indeed, these discrepancies could also be explained by the different half-life of these proliferative markers. PCNA has a reported half-life of 20 h, whereas Ki-67 has a much shorter half-life, which is only around 60–90 min *in vitro* (Bravo and MacDonald-Bravo, 1987; Bruno and Darzynkiewicz, 1992; Heidebrecht et al., 1996). In addition, cyclin D has a rather short half-life, which is less than 30 min *in vitro* (Diehl et al., 1997; Kanie et al., 2012). These findings suggest that collection of tissue samples should be done within a short period of time and PCNA could be a more stable proliferative marker as compared to Ki-67 and cyclin D. Furthermore, as reviewed by Bara et al. (2010) based on the report summarized by Lambert et al. (1993), the lack of staining of PCNA/Ki-67 in asthmatic airways could be due to the increased cellular proliferation that may have occurred prior to obtaining a biopsy. Nevertheless, compounding cellular dynamics such as ASM migration and/or diminished apoptosis in response to the inflammatory mediators could explain these variations.

## The Role of Inflammation on ASM Abundance

Numerous investigations have employed *in vitro* models to assess the role of inflammatory mediators in increasing ASM cell proliferation (Camoretti-Mercado, 2009; Salter et al., 2017). A wide range of inflammatory mediators and growth factors released during an asthma attack were reported to induce ASM cell proliferation *in vitro* (Halwani et al., 2011a; Stamatiou et al., 2012; Al Heialy et al., 2013; Lan et al., 2017). Among these mediators, IL-8, eotaxin, and MIP-1α have been shown to reduce the rate of apoptosis in human non-asthmatic ASM cells (Halwani et al., 2011a). Transforming growth factor beta-1 (TGF-β1) has been shown to be elevated in the asthmatic airway and its regulatory role in airway remodeling has been intensively reviewed (Redington et al., 1997; Vignola et al., 1997; Camoretti-Mercado and Solway, 2005; Makinde et al., 2007; Halwani et al., 2011b; Al-Alawi et al., 2014). TGF-β1 was reported to induce both ASM proliferation and migration (Li et al., 2015; Fang et al., 2018; Gao et al., 2018; Liu et al., 2019) and reduces ASM apoptosis as well (Fang et al., 2018; Gao et al., 2018). In a study that employed human non-asthmatic ASM cells microRNA-217 (miR-217) was shown to inhibit TGF-β1-induced ASM proliferation and migration but promoted apoptosis in TGF-β1-induced ASM through a similar signaling molecule, namely zinc finger E-box binding homeobox 1 (ZEB1) (Gao et al., 2018). Zhang et al. (2016a) demonstrated enhanced miR-146a expression inhibits epidermal growth factor receptor (EGFR) with suppression of proliferation and increased rates of apoptosis in human ASM cells. Similarly, miR-142 was demonstrated to inhibit proliferation and promote apoptosis in ASM cells isolated from asthmatic rats through a mechanism that involved TGF-β1-dependent EGFR signaling (Wang et al., 2018b). Furthermore, inhibitors of EGFR and p38 have been shown to inhibit migration of ASM cells from allergen sensitized and challenged mice (Wang et al., 2016). The role of p38 in mediating actin cytoskeleton remodeling in ASM cells is dependent upon heat shock protein 27 (HSP27) that acts as a downstream substrate for p38 and regulator of cytoskeletal remodeling in ASM (Hedges et al., 1999; An et al., 2004). These reports suggest a dynamic interrelationship between rates of ASM proliferation, apoptosis, and migration that could possibly be regulated through similar pathways.

## Signal Transduction Regulation in ASM Proliferation

ASM proliferates in response to growth factors, inflammatory cytokines, and contractile agonists that activate receptor tyrosine kinases (RTKs), G-protein coupled receptors (GPCRs) and non-receptor tyrosine kinases (nRTKs) (Lazaar and Panettieri, 2005). In addition, free fatty acids also activate both GPCRs and nRTKs to cause proliferation with a possible association between obesity and increased ASM mass in human non-asthmatic ASM (Matoba et al., 2018). Activation of RTKs and GPCRs stimulate the activation of Ras, a small guanosine triphosphatase (GTPase) protein, which is known to stimulate both mitogen-activated protein kinase (MAPK) and phosphoinositide 3-kinase (PI3K)/AKT pathways that promote ASM proliferation (Tliba and Panettieri, 2009). The three main MAPK signaling pathways; ERK, c-jun N-terminal kinase (JNK) and p38, as well as the PI3K/AKT signaling pathway are well-recognized as major mitogenic pathways involved in ASM proliferation (Lee et al., 2001; Fernandes et al., 2004; Krymskaya, 2007; Burgess et al., 2008; Halwani et al., 2011a; Chiba et al., 2017). Among these proliferation pathways, Walker et al. (1998) demonstrated that the PI3K pathway is the key signaling route in ASM proliferation while the ERK pathway provides a complementary signal required for the full mitogenic response in bovine non-asthmatic ASM cells. The inhibitor (LY294002) of the PI3K signaling pathway completely abolished PDGF-BB and thrombin-induced DNA synthesis in bovine non-asthmatic ASM cells, whereas the inhibitor of ERK (PD98002), at a concentration that completely inhibits ERK kinase activity, only partially inhibited DNA synthesis in bovine non-asthmatic ASM. Indeed it has been shown that in response to strong mitogenic stimuli, such as 10% foetal bovine serum (FBS), MAP kinase phosphatase-1 (MKP-1), a MAPK phosphatase, is up-regulated in human asthmatic ASM cells leading to a reduction of ERK activation and subsequently causing a shift of the mitogenic role to the PI3K pathway alone (Burgess et al., 2008). In response to induction with FBS it has been shown that both PI3K and ERK pathways are involved in non-asthmatic and asthmatic ASM cell proliferation, although strong stimuli (10% FBS) causes a reduction of ERK activation in inducing asthmatic ASM proliferation. These findings suggest that therapeutic intervention through targeted disruption of the PI3K pathway rather than the ERK pathway could be more effective in reversing or inhibiting increased ASM mass.

Whereas activation of Ras induces full activation of ERK and modest activation of stress-induced MAPKs (JNK and p38) in bovine ASM (Page et al., 1999a), Ras-related C3 botulinum toxin substrate 1 (Rac1), another small GTPase, is reported to induce full activation of JNK and p38 without any effect upon ERK activation in smooth muscle cells (Page et al., 1999a; Li et al., 2000). These findings suggest that both Ras-MAPKs and Rac-JNK/p38 pathways are involved in ASM proliferation with possibly varying influences. Upon activation of ERK, JNK and PI3K/AKT signaling pathways, expression of cyclin D1, a key cell cycle regulator, is up-regulated (Page et al., 2000; Ravenhall et al., 2000; Chiba et al., 2017), leading to ASM proliferation, whereas p38 induces proliferation in human ASM cells through the phosphorylation of retinoblastoma protein (pRb) instead of cyclin D1 (Fernandes et al., 2004). Furthermore, PDGF-induced activation of p38 in bovine ASM cells has been demonstrated to negatively regulate cyclin D1 expression leading to inhibition of ASM proliferation (Page et al., 2001). These contradictory findings on the role of p38 in ASM proliferation could be due to the nature of the mitogenic stimuli, a dual role of p38 and/or different species. A dual role of p38 in cellular proliferation has been reported in fibroblasts, in which the level of p38 activation in response to different stimuli determines either mitogen-induced proliferation or stress-induced cell cycle arrest as a cellular response (Faust et al., 2012). Furthermore, the mitogenic effect of p38 in inducing ASM proliferation has been demonstrated to be mitogen-specific in which basic fibroblast growth factor (bFGF), but not thrombin, induced p38 phosphorylation whereas both stimuli were equally effective in inducing pRb phosphorylation and subsequent ASM proliferation (Fernandes et al., 2004).

Recently the role of microRNA in regulation of major proliferative pathways signaling has been demonstrated. PI3K and JNK signaling pathways can be co-regulated through microRNA-708 in which its overexpression was shown to induce expression of PTEN, a tumor suppressor, resulting in a reduction of AKT phosphorylation, a downstream target in PI3K pathway in human ASM cells (Dileepan et al., 2014). Furthermore, microRNA-708 has also been shown to decrease the phosphorylation of JNK in human ASM cells and this effect was associated with an increased expression of MKP1 (Dileepan et al., 2014). In contrast, increased MKP1 expression was not associated with decreased p38 phosphorylation which is inconsistent with previous findings that MKP1 can dephosphorylate both JNK and p38 (Taylor et al., 2013; Dileepan et al., 2014). This could be due to compensatory upregulation of several upstream kinases involved in the phosphorylation of p38.

Several studies in various cell types have demonstrated convergence of both PI3K and JNK signaling pathways. A study in human bronchial epithelial cells involving overexpression of mutant p85 (PI3K) and dominant-negative AKT demonstrated that PI3K and AKT both act as upstream regulators of JNK and c-Jun, suggesting a PI3K/AKT/JNK/c-Jun/cyclin D signaling pathway in the regulation of cell proliferation (Ding et al., 2009). In HeLa cells the PI3K inhibitor wortmannin inhibited EGF-induced JNK activation while no inhibitory effect was detected following UV- or osmotic stress-induced JNK activation, suggesting PI3K acts as an upstream activator in mediating mitogenic-induced JNK activation (Logan et al., 1997). Conversely, JNK was reported to act as an upstream activator of AKT in serotonin (5-HT)-induced pulmonary artery smooth muscle cells (PASMCs), whereby inhibition of JNK was shown to suppress the activation of AKT, subsequently inhibiting PASMCs proliferation (Wei et al., 2010). Since, as discussed earlier, JNK is suggested to have other upstream activators in ASM cells and coupled with evidence of interplay between JNK and PI3K/AKT pathways in PASMCs and cancer cells, the existence of crosstalk between these two pathways in regulating ASM cell proliferation cannot be ruled out. Further investigations into the probable interplay between these two pathways in ASM proliferation would therefore yield crucial information for the development of more selective and safer drugs.

The JAK2/STAT3 pathway also plays a major role in ASM mitogenesis. This pathway was demonstrated to contribute to the mitogenesis of PDGF-induced ASM. Several cell cycle regulators such as cyclin D1, cyclin D3, and p27^Kip1^, Rac1 as well as c-myc, a proto-oncogene, are regulated through the activation of STAT3 in human ASM cells, thus inducing ASM proliferation (Simon et al., 2002; Simeone-Penney et al., 2008). The role of the transcription factor STAT3 in ASM proliferation was further confirmed with the use of STAT3-short hairpin RNA (shRNA) transfected ASM cells, whereby STAT3-shRNA fully abrogated the proliferative effect of IgE and PDGF upon ASM cells (Redhu et al., 2013). To date, studies have demonstrated that STAT3 can be phosphorylated by multiple upstream molecules which include RTKs, nRTKs, (Src and JAK2) as well as downstream kinases of MAPKs and PI3K in cancer cells (Bishop et al., 2014). Some of these findings were observed in human ASM cells as well, whereby nRTKs such as Src and JAK2, and RTK growth factor, such as PDGF, were reported to mediate STAT3 activation (Simon et al., 2002; Simeone-Penney et al., 2008). In addition to this, inhibition of ERK has also been demonstrated to inhibit IL-17-induced STAT3 phosphorylation in human ASM cells suggesting a functional role of ERK-STAT3 crosstalk (Saleh et al., 2009). Furthermore sh-RNA silencing of STAT3 has been demonstrated to completely abolish IgE- and PDGF-mediated proliferation in human ASM cells (Redhu et al., 2013). These observations suggest that STAT3 plays a major role downstream of most of the signaling pathways or receptors involved in ASM proliferation.

**Figure 1:**
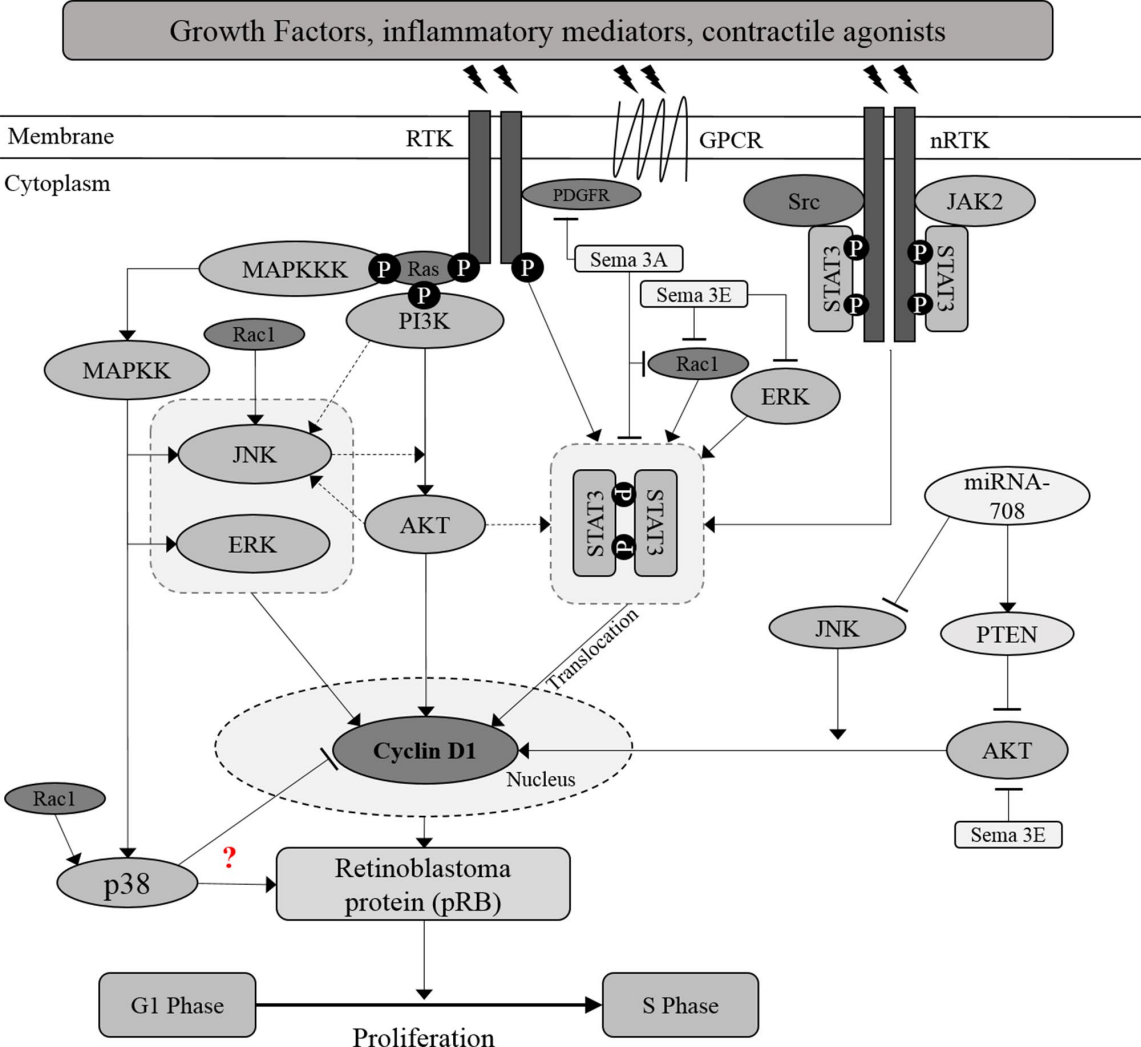
Crosstalk between proliferative signaling pathways in ASM. Inflammatory mediators, growth factors and contractile agonists released during asthma inflammation, induce ASM proliferation through activation of MAPKs, PI3K/AKT, and JAK2/STAT3 signaling pathways. AKT is suggested to act as the upstream activator of STAT3, a transcription factor. Besides, JNK may acts as the upstream activator or downstream target of AKT. Further investigation should be carried out to map out the association between AKT and JNK. Activation of these signaling molecules will increase the protein expression of cyclin D1 and subsequently induce the cell cycle progression. Solid arrows indicate the associations reported earlier while dotted arrows indicate the possible association that may occur, in which further examination need to be carried out.? Indicates the inconsistent findings.

In recent years, the role of semaphorins (Sema) as therapeutic targets in airway disease has been addressed (Movassagh et al., 2018). Semaphorins are originally recognised as neuronal chemorepellents and were reported to play a role in cellular activities, such as cell proliferation, migration, adhesion, and angiogenesis (Movassagh et al., 2017a). Increased AHR, airway remodeling and Th2/Th17 inflammation in chronic allergic airway disease were shown to be significantly augmented in a Sema 3E-deficient mouse model (Movassagh et al., 2017b) and therefore demonstrating an essential role of Sema 3E in suppressing hallmarks of chronic airway disease. Sema 3E and 3A expression and their receptors were reported to be expressed in both human asthmatic and non-asthmatic ASM. Further investigations revealed that PDGF-induced ASM proliferation can be inhibited with exogenous Sema 3E and 3A in both non-asthmatic and asthmatic ASM cells (Movassagh et al., 2014; Movassagh et al., 2016). The inhibition was reported to be associated with several key proliferative signaling molecules. It is possible that semaphorin inhibition of ASM proliferation could be through its effects upon PDGFR as an upstream molecule. Both Semaphorins 3A and 3E decrease Rac1 GTPase activity in ASM. Furthermore, Sema 3A decreases the phosphorylation of PDGF-induced PDGFR and STAT3 in both human non-asthmatic and asthmatic ASM cells while Sema 3E was reported to decrease the phosphorylation of AKT and ERK1/2 (Movassagh et al., 2014; Movassagh et al., 2016). Further work is needed to fully address the functional role of Sema 3E upon PDGFR. Crosstalk between signaling pathways in the regulation of ASM proliferation is illustrated in **Figure 1**.

## Signal Transduction Regulation in ASM Migration

Migration of ASM cells is accomplished through five main steps namely; cell polarization, protrusion, adhesion, contraction, and retraction. These steps are controlled by specific signaling molecules in response to stimuli such as growth factors, cytokines, chemokines, and the extracellular matrix (ECM) (Salter et al., 2017). Cellular migration is initiated *via* activation of RTKs, GPCRs, and matrix adhesive proteins, particularly integrins, which consequently activate remodeling of the cytoskeleton and rearrangement of subcellular organelles. Cytoskeletal remodeling is initiated with actin polymerization through activation of multiple signaling molecules which include Src, phospholipase C, c-Abl, phosphatidylinositol 4,5-bisphosphate (PIP_2_), and PI3K, leading to the activation of AKT, Rho, Ras, PKA, Cortactin, Cdc42, focal adhesion kinase (FAK), and myosin light chain kinase (MLCK) that in turn regulate cell polarization, actin polymerization, and traction force (Gerthoffer, 2008). This process will extend the leading edge (anterior) of a migrating cell protruding towards the stimuli through focal contacts and enhance attachment of the cell membrane to the ECM, while myosin motors will bind to actin filaments and generate traction force at the posterior end that moves the cell forward (Gerthoffer et al., 2012; Salter et al., 2017).

Various signaling molecules including FAK, PI3K, PTEN, ERK, p38, Src, Rho kinase, c-Abl, and Wnt/β-catenin have been reported to be involved in the regulation of ASM cell migration in response to migratory stimuli (Parameswaran et al., 2002; Parameswaran et al., 2004; Lan et al., 2010; Cleary et al., 2014; Fang et al., 2017). However, the exact regulatory mechanism involved remains unclear. FAK, a nRTK, has been recognized as the key signaling molecule implicated in integrin pathways which are known to regulate cytoskeletal organization during cell migration. FAK regulates ASM migration through its interaction with several signaling molecules including PI3K, Src and Rho kinase (Parameswaran et al., 2002; Parameswaran et al., 2004; Lan et al., 2010; Zhao and Guan, 2011; Cleary et al., 2014; Fang et al., 2017). Furthermore, FAK is reported to be co-regulated with phosphatidylinositol 3,4,5-trisphosphate (PIP_3_), a product of the PI3K signaling molecule, through PTEN. Overexpression of PTEN dephosphorylates both PIP_3_ and FAK, hence significantly inhibiting ASM migration. This suggests that PTEN may not only be involved in the regulation of ASM cell proliferation, but also in the regulation of ASM cell migration (Tamura et al., 1999; Lan et al., 2010).

Conflicting reports exist regarding the role of p38 in ASM cell migration. Using chemokines CXCL2 and CXCL3 as inducers, Al-Alwan et al. (2013) demonstrated that PI3K plays a major role in the migration of human asthmatic ASM cells whereas ERK and p38 were shown to affect the migration of normal ASM cells without any effect upon asthmatic ASM cells. However, upon activation with EGFR, p38 was demonstrated to be involved in asthmatic ASM cell migration while specific inhibitors of p38 and EGFR prevented cellular migration (Wang et al., 2016). These inconsistent findings may be due to the effects of different stimuli upon cellular migratory pathways. The role of p38 in mediating actin cytoskeleton remodeling in ASM cells is dependent upon heat shock protein 27 (HSP27) that acts as a downstream substrate for p38 and regulator of cytoskeletal remodeling in ASM (Hedges et al., 1999; An et al., 2004).

The nRTK c-Abelson tyrosine kinase (c-Abl) has been detected in the leading edge of human ASM cells (Cleary et al., 2014). Silencing of c-Abl inhibited adhesion-induced phosphorylation of cortactin and localization of profilin-1 (Pfn-1) at the cell edge. Pfn-1 is a key enzyme that catalyzes actin elongation and polymerization (Kiaei et al., 2018). Phosphorylated cortactin recruits Pfn-1 to the leading edge and promotes localized actin filament rearrangement, leading edge formation and cell migration. Inhibition of cortactin phosphorylation and Pfn-1 localization upon silencing of c-Abl results in the inhibition of ASM migration and therefore suggests that c-Abl is possibly involved in the regulation of smooth muscle migration *via* cortactin and Pfn-1. The role of Wnt/β-catenin has also been reported in the regulation of migration in ASM cells isolated from rats (Fang et al., 2017). Increased expression of both β-catenin mRNA and protein has been demonstrated in the ASM of chronic asthmatic rats. Silencing of β-catenin significantly inhibited ASM migration, suggesting that β-catenin may be involved in the regulation of ASM migration and the pathology of asthma.

Recent studies have demonstrated the role of the sonic hedgehog (SHH) pathway in smooth muscle cell (SMC) migration. Particulate matter 2.5 (PM2.5), which is particulate matter with an aerodynamic diameter smaller than 2.5 µm, has been shown to induce migration of human ASM cells *via* the SHH pathway (Ye et al., 2018). This pathway plays a major role in developmental processes (Chouldhry et al., 2014) and several studies have demonstrated that the activation of the SHH signaling pathway induces migration of various cell types including glioblastoma, liver cancer cells, fibroblasts, endothelial cells, monocytes as well as aortic SMCs (Dunaeva et al., 2010; Renault et al., 2010; Polizio et al., 2011; Chen et al., 2013; Yao et al., 2014; Chang et al., 2015). PDGF-BB-induced aortic SMC migration is reduced following inhibition of the SHH pathway suggesting a major role of this pathway in aortic SMC migration. Matrix metalloproteinases (MMPs), AKT, and ERK1/2 were demonstrated to play a significant role in inducing migration of human ASM cells (Nishihara-Fujihara et al., 2010) and furthermore in aortic SMC migration. Yao et al. (2014) demonstrated that recombinant SHH induces activation of AKT as well as ERK1/2. Activation of the SHH signaling pathway induced migration through the induction of AKT-mediated activation of MMP-2 and MMP-9 in both glioblastoma and liver cancer cells (Chen et al., 2013; Chang et al., 2015). Hence an understanding of the precise functional role of the SHH pathway in ASM migration is of great interest. The dearth of reports regarding the functional role of SHH pathway in ASM migration warrants further assessment and understanding of the role of this signaling pathway.

## Crosstalk Between Signal Transduction in ASM Proliferation and Migration

Multiple studies have demonstrated that both proliferation and migration of ASM cells can be co-regulated through similar signaling molecules or pathways. Proliferation signaling molecules such as STAT3 and JNK, and also cell cycle proteins such as cyclin D1, p27^Kip1^, and S-phase kinase associated protein-2 (Skp-2), were reported to play a regulatory role in ASM cell migration. Silencing of STAT3 with shRNA has been shown to completely abolish PDGF-induced human ASM cell migration (Redhu et al., 2013). Rac1, a small GTPase which is also known to promote cytoskeletal reorganization at the leading edge promotes directional cell migration in mouse embryonic fibroblasts through STAT3 regulation (Teng et al., 2009). In ASM cells, Rac1 also acts as an upstream activator of JNK as well as cyclin D1 in mediating PDGF-activated STAT3 induction of ASM growth (Page et al., 1999b; Simon et al., 2002). However, whether Rac1 plays a crucial role in STAT3-mediated ASM migration remains to be determined. The involvement of JNK in cellular migration has been reported in several cell types (Zeke et al., 2016; Zhang et al., 2016b). Increasing the expression of BRAF-activated noncoding RNA (BANCR) has been demonstrated to induce vascular smooth muscle cell (VSMC) migration through the activation of JNK (Li et al., 2017). Furthermore, inhibition of JNK activity of angiotensin II (Ang II)-induced rat aortic smooth muscle cells (RASMCs) (Nagayama et al., 2015) and serotonin-induced pulmonary artery smooth muscle cells (ASMCs) (Wei et al., 2010) suppresses cellular migration. Clearly more mechanistic studies are needed to explain the role of JNK and Rac1 in ASM cell migration.

The role of cell cycle proteins in cellular migration has been acknowledged in various experimental systems. The protein p27^Kip1^ has been shown to inhibit vascular smooth muscle cell migration (Sun et al., 2001) and also shown to regulate cell migration through the Rho kinase pathway in which p27^Kip1^ interferes with the interaction between RhoA and its activators (Besson et al., 2004). Indeed, the role of Rho kinase in migration of human non-asthmatic ASM cells has been previously demonstrated in which PDGF-induced ASM cell migration was significantly suppressed following treatment with the Rho kinase inhibitor Y27632 (Parameswaran et al., 2002). Furthermore, the Rho kinase pathway also regulates proliferation of human non-asthmatic ASM cells in which inhibitors of Rho or Rho-associated kinase were demonstrated to cause significant reduction in proliferation (Takeda et al., 2006; Ross et al., 2013).

As discussed earlier, FAK is known to regulate ASM migration. Apart from this role, Bond et al. (2004) demonstrated FAK involvement in smooth muscle cell proliferation. In their study Bond and colleagues demonstrated significant inhibition of Skp-2 expression in rat aortic smooth muscle cells transfected with a dominant negative FAK_Y39/F_ mutant. Furthermore, the activity of FAK was reported to be essential in maintaining the stability of Skp-2, thus inducing the progression of cell cycle. Skp-2, is a F-box protein that regulates ubiquitination and proteasome degradation and these findings strengthen the claim that Skp-2 is a downstream target of FAK (Bond et al., 2004). Apart from demonstrating the role of Skp-2 in regulating proliferation of rat aortic smooth muscle (Bond et al., 2004), Chen et al. (2014) described similar observations in human bladder smooth muscle cells. In both studies Skp-2 was demonstrated to regulate both rat aortic and human bladder smooth muscle proliferation through the degradation of p27^Kip1^ (Bond et al., 2004; Chen et al., 2014). It is possible that FAK could possibly induce the degradation of p27^Kip1^ by maintaining the stability of Skp-2, and subsequently induce smooth muscle cell proliferation.

**Figure 2:**
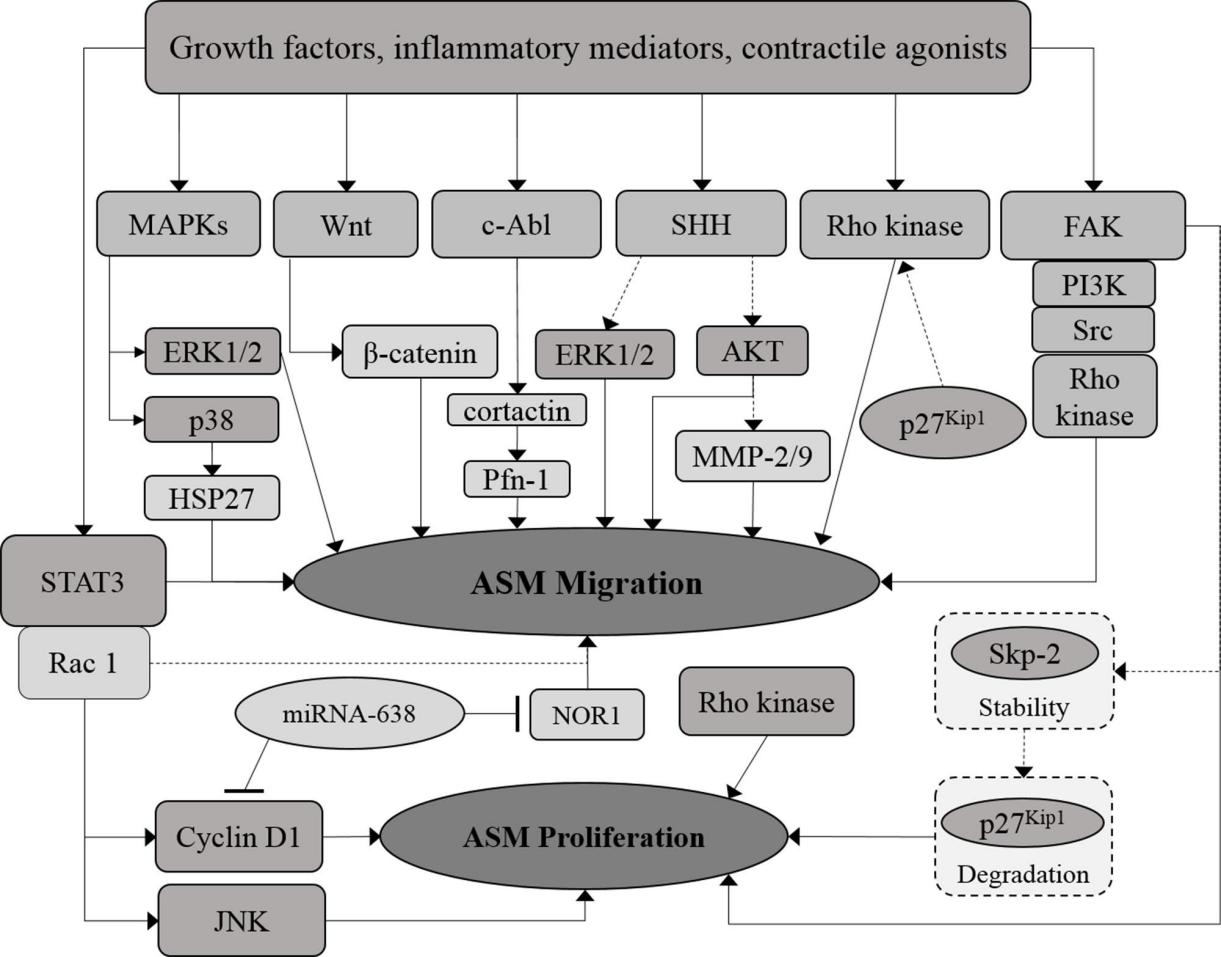
Crosstalk between proliferative and migratory signaling pathways in ASM. The proliferative and migration signaling molecules in ASM are co-related at certain points. MicroRNAs, which include miRNA-638, demonstrated to regulate both ASM proliferation and migration. Solid arrows indicate the associations reported earlier while dotted arrows indicate the possible association that may occur, in which further examination need to be carried out.

The potential of microRNAs to act as a regulator for both ASM cell proliferation and migration in allergic inflammation and asthma has been highlighted in recent years (Pua and Ansel, 2015; Munitz et al., 2016). MicroRNAs are non-coding RNA comprising ∼22 nucleotides that regulate gene transcription through binding to the 3′-untranslated region (UTR) of the targeted messenger RNAs (mRNAs) (Munitz et al., 2016). Overexpression of microRNA-638 has been demonstrated to inhibit human ASM cell proliferation and migration through direct targeting of cyclin D1 and neuron-derived orphan receptor 1 (NOR1) expression, whereas NOR1 has been shown to be crucial for human ASM cell migration (Wang et al., 2018a). The signaling molecules or pathways involved in ASM migration and the crosstalk between proliferative and migratory pathways in ASM are illustrated in the diagram below (**Figure 2**).

## Signal Transduction Regulation in ASM Apoptosis

The increase in number of ASM cells in the asthmatic airway is very much dependent upon the balance between cell proliferation and cell death. Apoptosis of ASM cells involves an intricate mechanism that counterbalances the accumulation of ASM mass during airway remodeling. Crosslinking of death receptors such as Fas, tumor necrosis factor receptor-1 (TNFR1), TNF-related apoptosis-inducing ligand receptor 1 (TRAIL-R1), and TRAIL-R2, with their respective ligands leads to apoptosis of proliferating human ASM cells (Hamann et al., 2000; Solarewicz-Madejek et al., 2009). Apoptosis is initiated *via* intrinsic and extrinsic pathways (Elmore, 2007). The intrinsic pathway can be activated by cellular stress that involves the activation of caspase-9 while the extrinsic pathway is activated by the death receptor which involves the activation of caspase-8 (Gerthoffer et al., 2012). ASM cell apoptosis can be mediated by both intrinsic and extrinsic pathways and a reduction in cell death has been suggested to contribute to the increased ASM mass. ASM cells isolated from a guinea pig model of asthma exhibited significantly higher survival signals as compared to normal control groups (Zhang et al., 2017b). These survival signals include higher expression of the anti-apoptotic protein Bcl-2 and lower expression of pro-apoptotic proteins as well as downstream effectors such as Bcl-associated X protein (BAX), caspase-9 and caspase-3. These survival signals could be contributors to the increased ASM mass as they reduce the expression level of apoptosis signaling molecules in ASM. However, the direct stimuli that induces these survival signals in ASM have not been defined. Several studies have revealed that ECM proteins and chemokines as well as cell–cell interaction during the inflammation process, reduces the rate of ASM apoptosis *in vitro*, hence inducing ASM remodeling in asthma (Freyer et al., 2001; Ramos-Barbon et al., 2005; Halwani et al., 2011a). Neutrophil elastase, an enzyme known to degrade ECM protein, has been shown to induce apoptosis in human non-asthmatic ASM cells (Oltmanns et al., 2005). This finding further confirmed that the ECM protein is crucial in maintaining ASM survival rate during inflammation. As mentioned earlier, the increased number of ASM cells is very much dependent on the balance between cell proliferation and cell apoptosis. Direct interaction between activated CD4^+^ T cell and ASM cell has been shown to induce ASM growth through the increase in proliferation and inhibition of apoptosis in ASM cells isolated from a rat model of asthma (Ramos-Barbon et al., 2005). In other systems, studies have demonstrated that not all proliferative stimuli reduce rates of ASM apoptosis. Growth factors, such as EGF, FGF, and PDGF, and pro-inflammatory mediators including bradykinin and histamine, have been shown to increase ASM mass through induction of ASM proliferation without any effect upon the rate of apoptosis in human non-asthmatic ASM cells (Freyer et al., 2001). It is possible that ASM proliferation and apoptosis could be regulated by distinct mechanisms but at the same time share common signaling molecules.

## Crosstalk Between Signal Transduction in ASM Proliferation and Apoptosis

ASM proliferation and apoptosis are interconnected through a web of cell cycle regulators and apoptosis stimuli (Amrani et al., 2001; Bai et al., 2010; Halwani et al., 2011a). MAPKs, JAK/STAT and PI3K/AKT signaling pathways, as well as a handful of microRNAs are reported to modulate both cell proliferation and apoptosis (Sui et al., 2014; Will et al., 2014; Sun et al., 2015; Severin et al., 2016). Epithelial-derived chemokines including eotaxin, RANTES, IL-8 and MIP-1β, were demonstrated to induce proliferation and reduce apoptosis in human non-asthmatic ASM cells. The anti-proliferative effect of these chemokines was found to be associated with the activation of ERK1/2 MAPK. However, the signaling pathway involved in these chemokines in reducing ASM apoptosis remains to be determined (Halwani et al., 2011a). On the other hand, the direct role of ERK1/2 upon ASM apoptosis has been reported by Bai et al. (2010), whereby inhibition of ERK (PD98059) increased the apoptosis percentage of ASM cells from allergen sensitized and challenged mice. This induction was found to be correlated with protein levels of BAX and caspase-3 and reduction of Bcl-2. Furthermore, TNFR1, a death receptor which is known to regulate apoptosis, was shown to promote ASM growth through activation of both p38 and ERK1/2 MAPKs in human non-asthmatic ASM cells (Amrani et al., 2001). These findings demonstrated that signaling molecules of the apoptosis pathway also regulate ASM proliferation. On the other hand, JNK inhibition using SP600125 had no effect upon ASM apoptosis in both human non-asthmatic and asthmatic ASM cells while the role of p38 upon ASM apoptosis was unclear (Chiba et al., 2017).

The role of microRNAs in regulating both ASM proliferation and apoptosis has been acknowledged in recent years. Overexpression of microRNA-142 and microRNA-146a, in rat ASM cells and human non-asthmatic ASM cells respectively, were shown to inhibit ASM proliferation and induce ASM apoptosis through direct targeting of EGFR (Zhang et al., 2016a; Wang et al., 2018b). In addition, microRNA-139-5p and microRNA-216a were demonstrated to exert similar effects in human non-asthmatic ASM cells *via* downregulation of brahma-related gene1 (Brg1) mRNA, a member of the switch/sucrose non-fermentable chromatin-remodeling complex and JAK2 respectively (Zhang et al., 2017a; Yan et al., 2018). These findings demonstrated that ASM proliferation and apoptosis can be co-regulated through EGFR, Brg1, and JAK2 with the aid of microRNA-142, microRNA-146a, microRNA-139-5p, and microRNA-216a.

The inhibitory effect of these microRNAs upon EGFR and Brg1 expression was demonstrated to reduce the activation or expression of proliferation-related signaling molecules and anti-apoptotic proteins while promoting pro-apoptotic proteins. Following upregulation of microRNA-142, expression of TGF-β, EGFR, AKT, phosphorylated-AKT, as well as anti-apoptotic proteins including Bcl-2 and B-cell lymphoma-extra-large (Bcl-xl) were reduced while expression of BAX and p21, a cyclin-dependent kinase inhibitor (CKI), were elevated (Wang et al., 2018b). On the other hand, microRNA-146a has been shown to reduce phosphorylation of ERK, STAT3, and EGFR and also expression of EGFR and Bcl-2, while promoting the activity of caspase 3/7 (Zhang et al., 2016a). Inconsistencies have been noted in the effects of microRNAs in which microRNA-139-5p has been reported to reduce AKT phosphorylation culminating in effects upon ASM proliferation and apoptosis (Zhang et al., 2017a) whereas Luo et al. (2013) observed reduced AKT phosphorylation only inhibited ASM proliferation without any effect upon apoptosis. Can AKT inhibition alone be sufficient to induce ASM apoptosis? Could microRNA-139-5p targeting other pro-apoptotic molecules be responsible for ASM apoptosis? Indeed more work is needed to further elucidate the role of AKT in ASM apoptosis.

**Figure 3:**
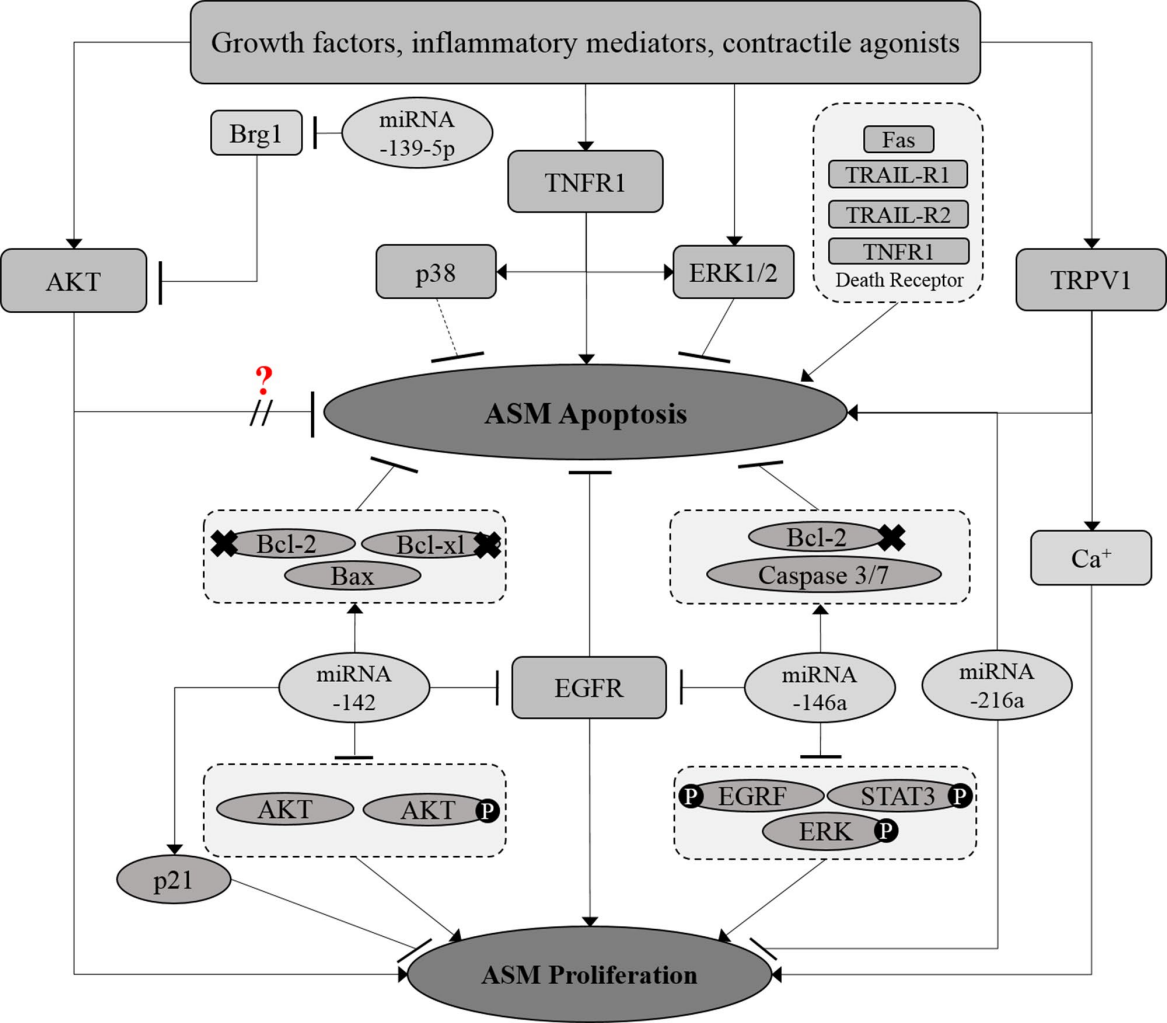
Crosstalk between proliferative and apoptotic signaling pathways in ASM. The proliferative and apoptosis signaling molecules in ASM are interrelated at certain points. MicroRNAs, which include miRNA-139-5p, miRNA-216a, and miRNA-142 demonstrated to co-regulate both ASM proliferation and survival. Solid arrows indicate the associations reported earlier while dotted arrows indicate the possible association that may occur, in which further examination need to be carried out. × Indicates the reduced expression level of the molecule.? Indicates the inconsistent findings.

The thermoreceptor, transient receptor potential vanilloid 1 (TRPV1) has also been reported to regulate ASM proliferation and apoptosis. Capsaicin, an agonist of the TRPV1 channel, has been shown to induce proliferation and inhibit apoptosis in rat asthmatic ASM cells while the TRPV1 antagonist capsazepine showed an opposite effect (Zhao et al., 2014). An increase in intracellular calcium ions following TRPV1 channel activation was suggested to be the cause of enhanced ASM proliferation however, the mechanism by which TRPV1 inhibits ASM apoptosis remains unclear. Elevated intracellular calcium ions has been linked to several cellular processes in ASM, including cell contractility, mobility, and proliferation (Berridge, 2008; Gerthoffer, 2008; Lipskaia et al., 2009) of which have been associated with the severity of airway hyperresponsiveness (Lauzon and Martin, 2016). As such, the role of the TRPV1 channel in reversing increased ASM mass warrants further investigation. Crosstalk between the signaling pathways involved in the regulation of ASM proliferation and apoptosis is illustrated in **Figure 3**.

## Conclusion

This communication highlights the signaling pathways and molecules involved in the regulation of ASM hyperplasia as well as the crosstalk that exists between these regulatory molecules. The role of signaling molecules upon ASM proliferation and migration in asthmatic versus non-asthmatic human ASM cells is summarized in **Table 1** and animal ASM cells *in vitro* or *in vivo* is summarized in **Table 2**. The signal transduction pathways and molecules involved in ASM hyperplasia are interrelated at certain points, however, there are still gaps and questions to be answered. The recent role of microRNAs in mediating ASM proliferation, migration and survival open up new avenues for a better understanding of tissue remodeling in asthma. As such, more work in this area is envisaged and the possible role of microRNAs as novel therapeutic targets is an interesting way forward. The use of bioinformatic tools in mapping out more detailed pathway interactions will further enhance our understanding of the complexity of smooth muscle responses in asthma and subsequently contribute to new approaches and novel drugs to control this disease.

**Table 1:** Summary of findings in studies using human asthmatic versus non-asthmatic ASM cells.

	Inducer	Treatment	Non-asthmatic ASM	Asthmatic ASM
(Burgess et al., 2008)	0.1% FBS	– – – ERK (U0126) PI3K (LY294002)	Proliferation ↑ p-ERK ↑ MKP-1 # Proliferation ↓ Proliferation ↓	Proliferation ↑ p-ERK ↑* MKP-1 # Proliferation ↓ Proliferation ↓
	1% FBS	– – – ERK (U0126) PI3K (LY294002)	Proliferation ↑ p-ERK ↑ MKP-1 ↑ Proliferation ↓ Proliferation ↓	Proliferation ↑* p-ERK ↑* MKP-1 ↑ Proliferation ↓ Proliferation ↓
	10% FBS	– – – ERK (U0126) PI3K (LY294002)	Proliferation ↑ p-ERK ↑* MKP-1 ↑ Proliferation ↓ Proliferation ↓	Proliferation ↑* p-ERK ↑ MKP-1 ↑* Proliferation # Proliferation ↓
(Movassagh et al., 2014)	PDGF	– Sema3E	Proliferation ↑ Proliferation ↓	Proliferation ↑ Proliferation ↓
(Movassagh et al., 2016)	PDGF	– Sema3A Sema3A Sema3A	Proliferation ↑ Proliferation ↓ p-GSK3β ↓ p-STAT3 ↓	Proliferation ↑ Proliferation ↓ p-GSK3β ↓ p-STAT3 ↓
(Al-Alwan et al., 2013)	CXCL2	– – – –	Migration ↑ p-p38 ↑ p-ERK ↑ p-PI3K #	Migration ↑* p-p38 # p-ERK # p-PI3K ↑
(Al-Alwan et al., 2013)	CXCL3	– – – –	Migration ↑ p-p38 ↑ p-ERK ↑ p-PI3K #	Migration ↑* p-p38 # p-ERK # p-PI3K ↑
(Chiba et al., 2017)	10% FBS	JNK (SP600125) JNK (SP600125) JNK (SP600125)	Proliferation ↓ Cyclin D1 ↓ Apoptosis #	Proliferation ↓ Cyclin D1 ↓ Apoptosis #

*↓* Indicates reduction; *↑* indicates increment; * indicates greater magnitude compared to the other group; # indicates no difference.

**Table 2:** Summary of findings in studies using non-human model systems.

Animal cells/models	Findings
Bovine ASM cells (Walker et al., 1998)	PI3K inhibitor (LY294002) completely abolished PDGF-BB and thrombine-induced DNA synthesis
Bovine ASM cells (Walker et al., 1998)	ERK inhibitor (PD98002) partially inhibited PDGF-BB and thrombin-induced DNA synthesis
Bovine ASM cells (Page et al., 1999a)	Ras induced full activation of ERK and modest activation of JNK and p38
Bovine ASM cells (Page et al., 2001)	PDGF-induced activation of p38 negatively regulated cyclin D1
Guinea pig asthma model (Zhang et al., 2017b)	Survival signals in ASM cells significantly elevated compared to control group
Mice asthmatic ASM cells (Bai et al., 2010)	ERK inhibitor (PD98059) increased the percentage of apoptotic cells in ASM
Rat asthma model (Fang et al., 2017)	Expression and protein level of β-catenin significantly elevated in ASM cells compared to control group
Rat asthmatic ASM cells (Fang et al., 2017)	Silencing of β-catenin significantly inhibited migration
Rat asthmatic ASM cells (Ramos-Barbon et al., 2005)	Direct interaction between activated CD4^+^ T cell and ASM cells induced ASM proliferation and inhibited ASM apoptosis
Rat asthmatic ASM cells (Zhao et al., 2014)	TRPV1 induced ASM proliferation and inhibited ASM apoptosis

## Author Contributions

HY, DI, HH, CT and MS contributed to the conceptual idea for the manuscript. HY and DI wrote the manuscript text with input from HY, DI, HH, CT and MS. HY prepared the figures. HY, DI, HH, CT and MS contributed to the critical review of the literature, editing of the manuscript text and review of the figures.

## Funding

This review was written as part of a literature search for experimental research upon pharmacological modulation of bronchial smooth muscle proliferation and migration that is financially supported by the Fundamental Research Grant Scheme of the Malaysian Ministry of Education (FRGS/1/2018/SKK10/UPM/01/1).

## Conflict of Interest

The authors declare that the research was conducted in the absence of any commercial or financial relationships that could be construed as a potential conflict of interest.

## References

[B1] Al Heialy S. Risse P. A. Zeroual M. A. Roman H. N. Tsuchiya K. Siddiqui S. (2013). T cell-induced airway smooth muscle cell proliferation *via the* epideral growth factor receptor. Am. J. Respir. Cell Mol. Biol. 49, 563–570. 10.1165/rcmb.2012-0356OC 23656597

[B2] Al-Alawi M. Hassan T. Chotirmall S.H. (2014). Transforming growth factor β and severe asthma: a perfect storm. Respir. Med. 108, 1409–1323. 10.16/j.rmed.2014.08.008 25240764

[B3] Al-Alwan L. A. Chang Y. Mogas A. Halayko A. J. Baglole C. J. Martin J. G. (2013). Differential roles of CXCL2 and CXCL3 ad their receptors in regulating normal and asthmatic airway smooth muscle cell migration. J. Immunol. 191, 2731–2741. 10.4049/jimmunol.1203421 23904157 PMC3748335

[B4] Al-Alwan L. A. Chang Y. Rousseau S. Martin J. G. Eidelman D. H. Hamid Q. (2014). CXCL1 inhibits airway smooth muscle cell migration through the decoy receptor Duffy antigen receptor for chemokines. J. Immunol. 193, 1416–1426. 10.4049/jimmunol.1302860 24981451

[B5] Amrani Y. Ammit A. J. Panettieri R. A. (2001). Tumor necrosis factor receptor (TNFR)1, but not TNFR2, mediates tumor necrosis factor-α-induced interleukin-6 and RANTES in human airway smooth muscle cells: role of p38 and p42/44 mitogen-activated protein kinases. Mol. Pharmacol. 60, 646–655. 11562425

[B6] An S. S. Fabry B. Mellema M. Bursac P. Gerthoffer W. T. Kayyali U. S. (2004). Role of heat shock protein 27 in cytoskeletal remodeling of the airway smooth muscle cell. J. Appl. Physiol. 97, 1701–1713. 10.1152/japplphysiol.01129.2003 14729728

[B7] Bai J. Liu X. S. Xu Y. J. Zhang Z. X. Xie M. Ni W. (2010). The effect of ERK signaling pathway on cell apoptosis in airway smooth muscle cells of chronic asthmatic rats. Cell Mol. Immunol. 26, 738–741. 20619097

[B8] Bara I. Ozier A. Tunon de Lara J. Marthan R. Berger P. (2010). Pathophysiology of bronchial smooth muscle remodelling in asthma. Eur. Respir. J. 36, 1174–1184. 10.1183/09031936.00019810 21037369

[B9] Baraldo S. Turato G. Bazzan E. Ballarin A. Damin M. Balestro E. (2011). Noneosinophilic asthma in children: relation with airway remodelling. Eur. Respir. J. 38, 575–583. 10.1183/09031936.00168210 21310879

[B10] Berair R. Saunders R. Brightling C. E. (2013). Origins of increased airway smooth muscle mass in asthma. BMC Med. 11, 1–6. 10.1186/1741-7015-11-145 23742314 PMC3688527

[B11] Berridge M. J. (2008). Smooth muscle cell calcium activation mechanism. J. Physiol. 586, 5047–5061. 10.1113/jphysiol.2008.160440 18787034 PMC2652144

[B12] Besson A. Gurian-West M. Schmidt A. Hall A. Roberts J. M. (2004). P27Kip1 modulates cell migration through the regulation of RhoA activation. Genes Dev. 18, 862–876. 10.1101/gad.1185504 15078817 PMC395846

[B13] Bishop J. L. Thaper D. Zoubeidi A. (2014). The multifaceted roles of STAT3 signaling progression of prostate cancer. Cancers (Basel) 6, 829–850. 10.3390/cancers6020829 24722453 PMC4074806

[B14] Blease K. Jakubzick C. Westwick J. Lukacs N. Kunkel S. L. Hogaboam C. M. (2001). Therapeutic effect of IL-13 immunoneutralization during chronic experimental fungal asthma. J. Immunol. 166, 5219–5224. 10.4049/jimmunol.166.8.5219 11290806

[B15] Bonacci J. V. Harris T. Wilson J. W. Stewart A. G. (2003). Collagen-induced resistance to glucocorticoid anti-mitogenic actions: a potential explanation of smooth muscle hyperplasia in asthmatic remodelled airway. Br. J. Pharmacol. 138, 1203–1206. 10.1038/sj.bjp.0705135 12711618 PMC1573763

[B16] Bond M. Sala-Newby G. B. Newby A. C. (2004). Focal adhesion kinase (FAK)-dependent regulation of S-phase kinase-associated protein-2 (Skp-2) stability. A novel mechanism regulating smooth muscle cell proliferation. J. Biol. Chem. 279, 37304–37310. 10.1074/jbc.M404307200 15208331

[B17] Bravo R. MacDonald-Bravo H. (1987). Existence of two population of cyclin/proliferating cell nuclear antigen during the cell cycle: association with DNA replication sites. J. Cell Biol. 105, 1549–1554. 10.1083/jcb.105.4.1549 2889739 PMC2114668

[B18] Bruno S. Darzynkiewicz Z. (1992). Cell cycle dependent expression and stability of the protein detected by Ki-67 antibody in HL-60 cells. Cell Prolif. 25, 31–40. 10.1111/j.1365-2184.1992.tb01435.x 1540682

[B19] Bullone M. Vargas A. Elce Y. Martin J. G. Lavoie J. (2017). Fluticasone/salmeterol reduces remodelling and neutrophilic inflammation in severe equine asthma. Sci. Rep. 7, 1–13. 10.1038/s41598-017-09414-8 28821845 PMC5562887

[B20] Burgess J. K. Lee J. H. Ge Q. Ramsay E. E. Poniris M. H. Parmentier J. (2008). Dual ERK and phosphatidylinositol 3-kinase pathways control airway smooth muscle proliferation differences in asthma. J. Cell. Physiol. 216, 673–679. 10.1002/jcp.21450 18338817

[B21] Busse W. W. (2010). The relationship of airway hyperresponsiveness and airway inflammation: airway hyperresponsiveness in asthma: its measurement and clinical significance. Chest 138, 4–10. 10.1378/chest.10-0100 PMC2914527 20668012

[B22] Camoretti-Mercado B. (2009). Targeting the airway smooth muscle for asthma treatment. Transl. Res. 154, 165–174. 10.1016/j.trsl.2009.06.008 19766960 PMC2764304

[B23] Camargo L. N. Righetti R. F. Aristoteles L.R.C.R.B. Santos T. M. Souza F. C. R. Fukuzaki S. (2018). Effects of anti-IL-17 on inflammation, remodeling, and oxidative stress in an experimental model of asthma exacerbated by LPS. Front. Immunol. 8, 1–14. 10.3389/fimmu.2017.01835 PMC5760512 29379497

[B24] Camoretti-Mercado B. Solway J. (2005). Transforming growth factor-beta1 and disorders of the lung. Cell Biochem. Biophys. 43, 131–148. 10.1385/CBB:43:1:131 16043890

[B25] Chang L. Zhao D. Liu H. B. Wang Q. S. Zhang P. Li C. L. (2015). Activation of sonic hedgehog signaling enhances cell migration and invasion by induction of matrix metalloproteinase-2 and -9 *via* phosphoinositide-3 kinase/AKT signaling pathway in glioblastoma. Mol. Med. Rep. 12, 6702–6710. 10.3892/mmr.2015.4229 26299938 PMC4626128

[B26] Chang Y. Al-Alwan L. Risse P. A. Halayko A. J. Martin J. G. Baglole C. J. (2012). Th17-associated cytokines promote human airway smooth muscle cell proliferation. FASEB J. 26, 5152–5160. 10.1096/fj.12-208033 22898922

[B27] Chang Y. Al-Alwan L. Risse P. A. Roussel L. Rousseau S. Halayko A. J. (2011). TH17 cytokines induce human airway smooth muscle cell migration. J. Allergy Clin. Immunol. 127, 1046–1053. 10.1016/j.jaci.2010.12.1117 21345484

[B28] Charriot J. Vachier I. Halinmi L. Gamez A. Boissin C. Salama M. (2016). Future treatment for asthma. Eur. Respir. Rev. 25, 77–92. 10.1183/16000617.0069-2015 26929425 PMC9487670

[B29] Chen J. S. Huang X. H. Wang Q. Huang J. Q. Zhang L. J. Chen X. L. (2013). Sonic hedgehog signaling pathway induces cell migration and invasion through focal adhesion kinase/AKT signaling-mediated activation of matrix metalloproteinase (MMP)-2 and MMP-9 in liver cancer. Carcinogenesis 34, 10–19. 10.1093/carcin/bgs274 22948179

[B30] Chen L. Wu T. Wei T. Wei X. Li S. Wang K. (2014). Skp2-mediated degradation of p27 regulates cell cycle progression in compressed human bladder smooth muscle cells. Kaohsiung J. Med. Sci. 30, 181–186. 10.1016/j.kjms.2013.07.002 24656158 PMC11915942

[B31] Chiba S. Okayasu K. Tsuchiya K. Tamaoka M. Miyazaki Y. Inase N. (2017). The c-jun N-terminal kinase signaling pathway regulates cyclin D1 and cell cycle progression in airway smooth muscle cell proliferation. Int. J. Clin. Exp. Med. 1092, 22552–22262.

[B32] Chouldhry Z. Rikani A. A. Choudhry A. M. Tariq S. Zakaria F. Asghar M. W. (2014). Sonic hedgedog signaling pathway: a complext network. Ann. Neurosci. 21, 28–31. 10.5214/ans.0972.7531.210109 25206052 PMC4117150

[B33] Cleary R. A. Wang R. Waqar O. Singer H. A. Tang D. D. (2014). Role of c-Abl tyrosine kinase in smooth muscle cell migration. Am. J. Physiol. Cell Physiol. 306, 753–761. 10.1152/ajpcell.00327.2013 PMC3989717 24477238

[B34] D’Amato G. Stanziola A. Sanduzzi A. Liccardi G. Salzillo A. Vitale C. (2014). Treating severe allergic asthma with anti-IgE monoclonal antibody (omalizumab): a review. Multidiscip. Respir. Med. 9, 1–6. 10.1186/2049-6958-9-23 24735949 PMC4113133

[B35] Dempsey O. (2000). Leukotriene receptor antagonist therapy. Postgrad. Med. J. 76, 767–773. 10.1136/pmk.76.902.767 11085767 PMC1741850

[B36] Denner D. R. Doeing D. C. Hogarth D. K. Dugan K. Naureckas E. T. White S. R. (2015). Airway inflammation after bronchial thermoplasty for severe asthma. Am. Thorac Soc. 12, 1302–1309. 10.1513/AnnalsATS.201502-082OC PMC5467092 26230374

[B37] Diehl J. A. Zindy F. Sherr C. J. (1997). Inhibition of cyclin D1 phosphorylation on threonine-286 prevents its rapid degradation *via the* ubiquitin-proteasome pathway. Genes Dev. 11, 957–972. 10.1101/gad.11.8.957 9136925

[B38] Dileepan M. Jude J. A. Rao S. P. Walseth T. F. Panettieri R. A. Subramanian S. (2014). MicroRNA-708 regulates CD38 expression through signaling pathways JNK MAP kinase and PTEN/AKT in human airway smooth muscle cells. Respir. Res. 15, 1–12. 10.1186/s12931-014-0107-0 25175907 PMC4156970

[B39] Ding J. Ning B. Huang Y. Zhang D. Li J. Chen C. (2009). PI3K/Akt/JNK/c-Jun signaling pathway is a mediator for arsenite-induced cyclin D1 expression and cell growth in human bronchial epithelial cells. Curr. Cancer Drug Targets 9, 500–509. 10.2174/156800909788486740 19519318 PMC3758122

[B40] Doeing D. C. Husain A. N. Naureckas E. T. White S. R. Hogarth D. K. (2013). Bronchial thermoplasty failure in severe persistent asthma: a case report. J. Asthma 50, 799–801. 10.3109/02770903.2013.796974 23651158 PMC3842589

[B41] Dombret M. Alagha K. Boulet L. P. Brillet P. Y. Joos G. Laviolette M. (2014). Bronchial thermoplasty: a new therapeutic option for severe, uncontrolled asthma in adults. Eur. Respir. J. 23, 510–518. 10.1183/09059180.00005114 PMC9487396 25445950

[B42] Dunaeva M. Voo S. van Oosterhoud C. Waltenberger J. (2010). Sonic hedgehog is a potent chemoattractant for human monocytes: diabetes mellitus inhibits Sonic hedgehog-induced monocyte chemotaxis. Basic Res. Cardiol. 105, 61–71. 10.1007/s00395-009-0047-x 19629560 PMC2789930

[B43] Elmore S. (2007). Apoptosis: a review of programmed cell death. Toxicol. Pathol. 35, 495–516. 10.1080/01926230701320337 17562483 PMC2117903

[B44] Fang P. Xue Y. Zhang Y. Fan N. Ou L. Leng L. (2018). SIRT7 regulates the TGF-β-induced proliferation and migration of mouse airway smooth muscle cells by modulating the expression of TGF-β receptor. Biomed. Pharnmacother. 104, 781–787. 10.1016/j.biopha.2018.05.060 29843083

[B45] Fang X. Zhao J. Shen L. Zheng M. Zhou H. Tao G. (2017). Role of Wnt/β-catenin signaling pathway in migration of asthmatic rat airway smooth muscle cells. Biomed. Res. 28, 7237–7242.

[B46] Faust D. Schmitt C. Oesch F. Oesch-Bartlomowicz B. Schreck I. Weiss C. (2012). Differential p38-dependent signalling in response to cellular stress and mitogenic stimulation in fibroblasts. Cell Commun. Signal. 10, 1–13. 10.1186/1478-811X-10-6 22404972 PMC3352310

[B47] Fehrenbach H. Wagner C. Wegmann M. (2017). Airway remodeling in asthma: what really matters. Cell Tissue Res. 367, 551–569. 10.1007/s00441-016-2566-8 28190087 PMC5320023

[B48] Fernandes D. Guida E. Koutsoubos V. Harris T. Vadiveloo P. Wilson J. W. (1999). Glucocorticoids inhibit proliferation, cyclin D1 expression, and retinoblastoma protein phosphorylation, but not activity of the extracellular-regulated kinases in human cultured airway smooth muscle. Am. J. Respir. Cell Mol. Bio. 21, 77–88. 10.1165/ajrcmb.21.1.3396 10385595

[B49] Fernandes D. J. Ravenhall C. E. Harris T. Tran T. Vlahos R. Stewart A. G. (2004). Contribution of the p38MAPK signalling pathway to proliferation in human airway smooth muscle cells is mitogen-specific. Br. J. Pharmacol. 142, 1182–1190. 10.1038/sj.bjp.0705809 15249425 PMC1575175

[B50] Freyer A. M. Johnson S. R. Hall I. P. (2001). Effects of growth factors and extracellular matrix on survival of human airway smooth muscle cells. Am. J. Respir. Cell Mol. Biol. 25, 569–576. 10.1165/ajrcmb.25.5.4605 11713098

[B51] Gao J. Cai F. Peng M. Wang B. (2013). Montelukast improves air trapping, not airway remodeling, in moderate-to-severe patients with asthma: A pilot study. Chin. Med. J. (Engl) 126, 2229–2234. 23786930

[B52] Gao Y. Wang B. Luo H. Zhang Q. Xu M. (2018). miR-217 repress TGF-β1-induced airway smooth muscle cell proliferation and migration through targeting ZEB1. Biomed. Pharmacother. 108, 27–35. 10.1016/j.biopha.2018.09.030 30212709

[B53] Ge Q. Poniris M. H. Moir L. M. Black J. L. Burgess J. K. (2012). Combined beta-agonists and corticosteroids do not inhibit extracellular matrix protein production *in vitro* . J. Allergy 2012, 1–7. 10.1155/2012/403059 PMC3303634 22500185

[B54] Gerthoffer W. T. Schaafsma D. Sharma P. Ghavami S. Halayko A. (2012). Motility, survival and proliferation. Compr. Physiol. 2, 255–281. 10.1002/cphy.c110018 23728975 PMC4120710

[B55] Gerthoffer W. T. (2008). Migration of airway smooth muscle cells. Proc. Am. Thorac. Soc. 5, 87–105. 10.1513/pats.200704-051VS PMC2645306 18094091

[B56] Goldstein R. H. Poliks C. F. Pilch P. F. Smith B. D. Fine A. (1989). Stimulation of collagen formation by insulin and insulin-like growth І in cultures of human lung fibroblasts. Endocrinology 124, 964–970. 10.1210/endo-124-2-964 2463909

[B57] Gosens R. Bos I. S. Zaagsma J. Meurs H. (2005). Protective effects of tiotropium bromide in the progression of airway smooth muscle remodeling. J. Respir. Crit. Care Med. 171, 1096–1102. 10.1164/rccm.200409-1249OC 15695490

[B58] Gu Y. Qi M. Li H. (2017). Th17 cells and their cytokines in the asthmatic airway remodeling: what do we know? J. Immunol. Infect. Inflam. Dis. 2, 1–5.

[B59] Halwani R. Al-Abri J. Beland M. Al-Jahdali H. Halayko A. J. Lee T. H. (2011a). CC and CXC chemokines induce airway smooth muscle proliferation and survival. J. Immunol. 186, 4156–4163. 10.4049/jimmunol.1001210 21368236

[B60] Halwani R. Al-Muhsen S. Al-Jahdali H. Hamid Q. (2011b). Role of transforming growth factor-β in airway remodeling in asthma. Am. J. Respir. Cell Mol. Biol. 44, 127–133. 10.1165/rcmb.2010-0027TR 20525803

[B61] Hamann K. J. Vieira J. E. Halayko A. J. Dorscheid D. White S. R. Forsythe S. (2000). Fas cross-linking induces apoptosis in human airway smooth muscle cells. Am. J. Physiol. Lung Cell Mol. Physiol. 278, 618–623. 10.1152/ajplung.2000.278.3.L618 10710535

[B62] Hassan M. Jo T. Risse P. A. Tolloczko B. Lemiere C. Olivenstein R. (2010). Airway smooth muscle remodeling is a dynamic process in severe long-standing asthma. J. Allergy Clin. Immunol. 125, 1037–1045. 10.1016/j.jaci.2010.02.031 20451038

[B63] Hedges J. C. Dechert M. A. Yamboliev I. A. Martin J. L. Hickey E. Weber L. A. (1999). A role for p38MAPK/HSP27 pathway in smooth muscle cell migration. J. Biol. Chem. 274, 24211–24219. 10.1074/jbc.274.34.24211 10446196

[B64] Heidebrecht H. J. Buck F. Haas K. Wacker H. H. Parwaresch R. (1996). Monoclonal antibodies Ki-S3 and Ki-S5 yield new data on the ‘Ki-67’ proteins. Cell Prolif. 29, 413–425. 10.1111/j.1365-2184.1996.tb00984.x 8883465

[B65] Hershenson M. B. Brown M. Camoretti-Mercado B. Solway J. (2008). Airway smooth muscle in asthma. Annu. Rev. Pathol. 3, 523–555. 10.1146/annurev.pathmechdis.1.110304.100213 18039134

[B66] Hoshino M. Ohtawa J. (2012). Effects of adding omalizumab, an anti-immunoglobulin E antibody, on airway wall thickening in asthma. Respir. 83, 520–528. 10.1159/000334701 22236804

[B67] Ijpma G. Panariti A. Lauzon A. M. Martin J. G. (2017). Directional preference of airway smooth muscle mass increase in human asthmatic airways. Am. J. Physiol. Lung Cell. Mol. Physiol. 312, 845–854. 10.1152/ajplung.00353.2016 28360113

[B68] James A. L. Elliot J. G. Jones R. L. Carroll M. L. Mauad T. Bai T. R. (2012). Airway smooth muscle hypertrophy and hyperplasia in asthma. Am. J. Respir. Crit. Care Med. 185, 1058–1064. 10.1164/rccm.201110-1849OC 22403800

[B69] Kanie T. Onoyama I. Matsumoto A. Yamada M. Nakatsumi H. Tateishi Y. (2012). Genetic reevaluation of the role of F-box proteins in cyclin D1 degradation. Mol. Cell. Biol. 32, 590–605. 10.1128/MCB.06570-11 22124152 PMC3266600

[B70] Kawaguchi M. Fujita J. Kokubu F. Ohara G. Huang S. K. Matsukura S. (2010). Induction of insulin-like growth factor-І by interleukin -17F in bronchial epithelial cells. Allergy 40, 1036–1043. 10.1111/j.1365-2222.2010.03527.x 20642578

[B71] Keglowich L. F. Borger P. (2015). The three A’s in asthma — Airway smooth muscle, airway remodeling & angiogenesis. Open Respir. Med. J. 9, 70–80. 10.2174/1874306401509010070 26106455 PMC4475688

[B72] Khan M. A. (2013). Inflammation signals airway smooth muscle cell proliferation in asthma pathogenesis. Multidiscip. Respir. Med. 8, 1–5. 10.1186/2049-6958-8-11 23388501 PMC3568740

[B73] Kiaei M. Balasubramaniam M. Kumar V. G. Reis R. J. S. Moradi M. Varughese K. I. (2018). ALS-causing mutations in profilin-1 alter its conformational dynamics: a computational approach to explain propensity for aggregation. Sci. Rep. 8, 1–10. 10.1038/s41598-018-31199-7 30166578 PMC6117255

[B74] Krymskaya V. P. (2007). Targeting the phosphatidylinositol 3-kinase pathway in airway smooth muscle: rationale and promise. BioDrugs 21, 85–95. 10.2165/00063030-200721020-00003 17402792

[B75] Lambert R. K. Wiggs B. R. Kuwano K. Hogg J. C. Pare P. D. (1993). Functional significance of increased airway smooth muscle in asthma and COPD. J. Appl. Physiol. 74, 2771–2781. 10.1152/jappl.1993.74.6.2771 8365980

[B76] Lan H. Wang N. Chen Y. Wang X. Gong Y. Qi X. (2017). Macrophage migration inhibitory fator (MIF) promotes rat airway muscle cell proliferation and migration mediated by ERK1/2 and FAK signaling. Cell Biol. Int. 42, 75–83. 10.1002/cbin.10863 28851074

[B77] Lan H. Zhong H. Gao Y. Ren D. Chen L. Zhang D. (2010). The PTEN tumor suppressor inhibits human airway smooth muscle cell migration. Int. J. Mol. Med. 26, 893–899. 10.3892/ijmm_00000539 21042784

[B78] Lauzon A. Martin J. G. (2016). Airway hyperresponsiveness; smooth muscle cell as the principal actor. F1000Res. 5, 1–9. 10.12688/f1000research.7422.1 PMC4786946 26998246

[B79] Lazaar A. L. Panettieri R. A. Jr. (2005). Airway smooth muscle: a modulator of airway remodeling in asthma. J. Allergy Clin. Immunol. 116, 488–495. 10.1016/j.jaci.2005.06.030 16159613

[B80] Lee J. H. Johnson P. R. Roth M. Hunt N. H. Black J. L. (2001). ERK activation and mitogenesis in human airway smooth muscle cells. Am. J. Physiol. Lung Cell. Mol. Physiol. 280, 1019–1029. 10.1152/ajplung.2001.280.5.L1019 11290527

[B81] Li C. Hu Y. Sturm G. Wick G. Xu Q. (2000). Ras/Rac-dependent activation of p38 mitogen-activated protein kinases in smooth muscle cells stimulated by cyclic strain stress. Arterioscler. Thromb. Vasc. Biol. 20, e1–e9. 10.1161/01.ATV.20.3.e1 10712420

[B82] Li H. Liu X. Zhang L. Li X. (2017). LncRNA BANCR facilitates vascular smooth muscle cell proliferation and migration through JNK pathway. Oncotarget 8, 114568–114575. 10.18632/oncotarget.21603 29383102 PMC5777714

[B83] Li L. Lu B. Wu H. Zhang H. Yao F. (2015). Apigenin inhibits TGF-β1-induced proliferation and migration of airway smooth muscle cells. Int. J. Clin. Exp. Pathol. 8, 12557–12563. 26722444 PMC4680389

[B84] Lipskaia L. Hulot J. S. Lompre A. M. (2009). Role of sarco/endoplasmic reticulum calcium content and calcium ATPase activity in the control of cell growth and proliferation. Pflugers Arch. 457, 673–685. 10.1007/s00424-007-0428-7 18188588

[B85] Liu Y. Sun X. Zhang Y. Wu H. Wang H. Yang R.(2019). Protocatechuic acid inhibits TGF-β-induced proliferation and migration of human airway smooth muscle cells. J. Pharmacol. Sci. 139, 9–14. 10.1016/j.jphs.2018.10.011 30472056

[B86] Lloyd C. M. Robinson D. S. (2007). Allergen-induced airway remodelling. Eur. Respir. J. 29, 1020–1032. 10.1183/09031936.00150305 17470623 PMC3384680

[B87] Logan S. K. Falasca M. Hu P. Schlessinger J. (1997). Phosphatidylinositol 3-kianse mediates epidermal growth factor-induced activation of the c-Jun N-terminal kinase signalling pathway. Mol. Cell. Biol. 17, 5784–5790. 10.1128/MCB.17.10.5784 9315636 PMC232426

[B88] Luo L. Gong Y. Q. Qi X. Lai W. Lan H. Luo Y. (2013). Effect of tumor suppressor PTEN gene on apoptosis and cell cycle of human airway smooth muscle cells. Mol. Cell. Biochem. 375, 1–9. 10.1007/s11010-012-1484-7 23275086

[B89] Maga G. Hubscher U. (2003). Proliferating cell nuclear antigen (PCNA): a dancer with many partners. J. Sci. 116, 3051–3060. 10.1242/jcs.00653 12829735

[B90] Makinde T. Murphy R. F. Agrawal D. K. (2007). The regulatory role of TGF-β in airway remodelling in asthma. Immunol. Cell Biol. 85, 348–356. 10.1038/sj.icb.7100044 17325694

[B91] Malmstrom K. Pelkonen A. S. Makela M. J. (2013). Remodeling, inflammation and airway responsiveness in early childhood asthma. Curr. Opin. Allergy Clin. Immunol. 13, 203–210. 10.1097/ACI.0b013e32835e122c 23339936

[B92] Maria D. Carolina V. Antonio M. Maurizia L. Gennaro D. (2017). Anticholinergic drugs in asthma therapy. Curr. Opin. Pulm. Med. 23, 103–108. 10.1097/MCP.0000000000000344 27820743

[B93] Martin J. G. Lauzon A. (2016). Airway hyperresponsiveness; smooth muscle as the principal actor. F1000Res. 5, 1–9. 10.12688/f1000research.7422.1 PMC4786946 26998246

[B94] Matoba A. Matsuyama N. Shibata S. Masaki E. Emala C. W. Sr. Mizuta K. (2018). The free fatty acid receptor 1 promotes airway smooth muscle cell proliferation through MEK/ERK and PI3K/Akt signalling pathways. Am. J. Physiol. Lung Cell. Mol. Physiol. 314, 333–348. 10.1152/ajplung.00129.2017 PMC5900353 29097424

[B95] Menzella F. Lusuardi M. Galeone C. Facciolongo N. (2017). Bronchial thermoplasty and the role of airway smooth muscle: are we on the right direction? Ther. Clin. Risk Manag. 13, 1213–1221. 10.2147/TCRM.S144604 29033571 PMC5614744

[B96] Meurs H. Gosens R. Zaagsma J. (2008). Airway hyperresponsiveness in asthma: lessons from in vitro model systems and animal models. Eur. Respir. J. 32, 487–502. 10.1183/09031936.00023608 18669789

[B97] Molet S. Hamid Q. Davoine F. Nutku E. Taha R. Page N. (2001). IL-17 is increased in asthmatic airways and induces human bronchial fibroblasts to produce cytokine. J. Allergy Clin. Immunol. 108, 430–438. 10.1067/mai.2001.117929 11544464

[B98] Movassagh H. Khadem F. Gounni A. S. (2018). Semaphorins and their roles in airway biology: potential therapeutic targets. Am. J. Respir. Cell Mol. Biol. 58, 21–27. 10.1165/rcmb.2017-0171TR 28817310

[B99] Movassagh H. Shan L. Duke-Cohan J. S. Halayko A. J. Uzonna J. E. Gounni A. S. (2017a). Semaphorin 3E alleviates hallmarks of house dust mite-induced allergic airway disease. Am. J. Pathol. 187, 1566–1576. 10.1016/j.ajpath.2017.03.008 28634005

[B100] Movassagh H. Shan L. Halayko A. J. Roth M. Tamm M. (2014). Neuronal chemorepellent semaphorin 3E inhibits human airway smooth muscle cell proliferation and migration. J. Allergy Clin. Immunol. 133, 560–567. 10.1016/j.jaci.2013.06.011 23932461

[B101] Movassagh H. Shan L. Duke-Cohan J. S. Chakir J. Halayko A. J. Koussin L. (2017b). Downregulation of semaphoring 3E promotes hallmarks of experimental chronic allergic asthma. Oncotarget 8, 98953–98963. 10.18632/oncotarget.22144 29228740 PMC5716780

[B102] Movassagh H. Tatari N. Shan L. Koussih L. Alsubait D. Khattabi M. (2016). Human airway smooth muscle cell proliferation from asthmatics is negatively regulated by semaphorin 3A. Oncotarget 7, 80238–80251. 10.18632/oncotarget.12884 27791986 PMC5348316

[B103] Moynihan B. J. Tolloczko B. Bassam S. E. Ferraro P. Michoud M. Martin J. G. (2008). IFN-γ, IL-4 and IL-13 modulate responsiveness of human airway smooth muscle cells to IL-13. Respir. Res. 9, 1–16. 10.1186/1465-9921-9-84 19116009 PMC2628656

[B104] Munitz A. Karo-Atar D. Foster P. S. (2016). Asthma diagnosis: MicroRNAs to the rescue. J. Allergy Clin. Immunol. 137, 1447–1448. 10.1016/j.jaci.2016.02.013 27155036

[B105] Nagayama K. Kyotani Y. Zhao J. Ito S. Ozawa K. Bolstad F. A. (2015). Exendin-4 prevents vascular smooth muscle cell proliferation and migration by angiotensin II *via* the inhibition of ERK and JNK signalling pathways. PLoS ONE 10, e0137960. 10.1371/journal.pone.0137960 26379274 PMC4574935

[B106] Naveed S. Clements D. Jackson D. J. Philp C. Billington C. K. Soomro I. (2017). Matrix metalloproteinase-1 activation contributes to airway smooth muscle growth and asthma severity. Am. J. Respir. Crit. Care Med. 195, 1000–1009. 10.1164/rccm.201604-0822OC 27967204 PMC5422648

[B107] Nishihara-Fujihara M. Shoji S. Maeda I. Shimoda T. Nishima S. Okamoto K. (2010). Involvement of fibronection and matrix metalloproteinases in airway smooth muscle cell migration for the process of airway remodelling. Allergol. Int. 59, 267–275. 10.2332/allergolint.09-OA-0153 20495339

[B108] Noveral J. P. Bhala A. Hintz R. L. Grunstein M. M. Cohen P. (1994). Insulin-like growth factor axis in airway smooth muscle cells. Am. J. Physiol. 267, 761–765. 10.1152/ajplung.1994.267.6.L761 7528983

[B109] Oliver M. N. Fabry B. Marinkovic A. Mijailovich S. M. Butler J. P. Fredberg J. J. (2007). Airway hyperresponsiveness, remodeling, smooth muscle mass. Right answer, wrong reason? Am. J. Respir. Cell Mol. Biol. 37, 264–272. 10.1165/rcmb.2006-0418OC 17463392 PMC1994228

[B110] Oltmanns U. Sukkar M. B. Xie S. John M. Chung K. F. (2005). Induction of human airway smooth muscle apoptosis by neutrophils and neutrophil elastase. Am. J. Respir. Cell Mol. Biol. 32, 334–341. 10.1165/rcmb.2004-0321OC 15653931

[B111] Ozier A. Allard B. Bara I. Girodet P. Trian T. Marthan R. (2011). The pivotal role of airway smooth muscle in asthma pathophysiology. J. Allergy (Cairo) 2011, 1–20. 10.1155/2011/742710 PMC3246780 22220184

[B112] Page K. Li J. Hershenson M. B. (2001). P38 MAP kinase negatively regulates cyclin D1 expression in airway smooth muscle cells. Am. J. Physiol. Lung Cell. Mol. Physiol. 280, 955–964. 10.1152/ajplung.2001.280.5.L955 11290520

[B113] Page K. Li J. Hershenson M. B. (1999a). Platelet-derived growth factor stimulation of mitogen-activated protein kinases and cyclin D1 promoter activity in culture airway smooth muscle cells. Am. J. Respir. Cell Mol. Biol. 20, 1294–1302. 10.1165/ajrcmb.20.6.3597 10340949

[B114] Page K. Li J. Hodge J. A. Liu P. T. Hoek T. L. V. Becker L. B. (1999b). Characterization of a Rac1 signaling pathway to cyclin D1 expression in airway smooth muscle cells. J. Biol. Chem. 274, 22065–22071. 10.1074/jbc.274.31.22065 10419534

[B115] Page K. Li J. Wang Y. Kartha S. Pestell R. G. Hershenson M. B. (2000). Regulation of cyclin D (1) expression and DNA synthesis by phosphatidylinositol 3-kinase in airway smooth muscle cells. Am. J. Respir. Cell Mol. Biol. 23, 436–443. 10.1165/ajrcmb.23.4.3953 11017907

[B116] Pain M. Bermudez O. Lacoste P. Royer P.-J. Botturi K. Tissot A. (2014). Tissue remodelling in chronic bronchial disease: from the epithelial to mesenchymal phenotype. Eur. Respir. Rev. 23, 118–130. 10.1183/09059180.00004413 24591669 PMC9487272

[B117] Panettieri R.A. Jr. (2004). Effects of corticosteroids on structural cells in asthma and chronic obstructive pulmonary disease. Proc. Am. Thorac. Soc. 1, 231–234. 10.1513/pats.200402-021MS 16113439

[B118] Parameswaran K. Cox G. Radford K. Janssen L. J. Sehmi R. O’Byrne P. M. (2002). Cysteinyl leukotrienes promote human airway smooth muscle migration. Am. J. Respir. Crit. Care Med. 166, 738–742. 10.1164/rccm.200204-291OC 12204874

[B119] Parameswaran K. Radford K. Fanat A. Stephen J. Bonnans C. Levy B. D. (2007). Modulation of human airway smooth muscle migration by lipid mediators and Th-2 cytokines. Am. J. Respir. Cell Mol. Biol. 37, 240–247. 10.1165/rcmb.2006-0172OC 17431098

[B120] Parameswaran K. Radford K. Zuo J. Janssen L. J. O’Byrne P. M. Cox P. G. (2004). Extracellular matrix regulates human airway smooth muscle migration. Eur. Respir. J. 24, 545–551. 10.1183/09031936.04.00113103 15459131

[B121] Polizio A. H. Chinchilla P. Chen X. Kim S. Manning D. R. Riobo N. A. (2011). Heterotrimeric Gi proteins link Hedgehog signalling to activation of Rho small GTPases to promote fibroblast migration. J. Biol. Chem. 286, 19589–19596. 10.1074/jbc.M110.197111 21474452 PMC3103338

[B122] Postma D. S. Kerstjens A. M. (1998). Characteristics of airway hyperresponsiveness in asthma and chronic obstructive pulmonary disease. Am. J. Respir. Crit. Care Med. 158, 187–192. 10.1164/ajrccm.158.supplement_2.13tac170 9817744

[B123] Pretolani M. Dombret M. C. Thabut G. Knap D. Hamidi F. Debray M. P. (2014). Reduction of airway smooth muscle mass by bronchial thermaplasty in patients with severe asthma. Am. J. Respir. Crit. Care Med. 190, 1452–1454. 10.1164/rccm.201407-1374LE 25496106

[B124] Pua H. H. Ansel K. M. (2015). MicroRNA regulation of allergic inflammation and asthma. Curr. Opin. Immunol. 36, 101–108. 10.1016/j.coi.2015.07.006 26253882 PMC4593751

[B125] Ramos-Barbon D. Fraga-Iriso R. Brienza N. S. Montero-Martinez C. Verea-Hernando H. Olivenstein R. (2010). T cells localize with proliferating smooth muscle alpha-actin+cell compartments in asthma. Am. J. Respir. Crit. Care Med. 182, 317–324. 10.1164/rccm.200905-0745OC 20395563

[B126] Ramos-Barbon D. Presley J. F. Hamid Q. A. Fixman E. D. Martin J. G. (2005). Antigen-specific CD4+ T cells drive airway smooth muscle remodeling in experimental asthma. J. Clin. Invest. 115, 1580–1589. 10.1172/JCI19711 15902312 PMC1088014

[B127] Ravenhall C. Guida E. Harris T. Koutsoubos V. Stewart A. (2000). The importance of ERK activity in the regulation of cyclin D1 levels and DNA synthesis in human cultured airway smooth muscle. Br. J. Pharmacol. 131, 17–28. 10.1038/sj.bjp.0703454 10960064 PMC1572283

[B128] Redhu N. S. Shan L. Al-Subait D. Ashdown H. L. Movassagh H. Lamkhioued B. (2013). IgE induces proliferation in human airway smooth muscle cells: role of MAPK and STAT3 pathways. Allergy Asthma Clin. Immunol. 9, 1–10. 10.1186/1710-1492-9-41 24499258 PMC3842672

[B129] Redington A. E. Madden J. Frew A. J. Djukanovic R. Roche W. R. Holgate S. (1997). Transforming growth factor-β1 in asthma. Measurement in bronchoalveolar lavage fluid. Am. J. Respir. Crit. Care Med. 156, 642–647. 10.1164/ajrccm.156.2.9605065 9279252

[B130] Renault M. A. Roncalli J. Tongers J. Thorne T. Klyachko E. Misener S. (2010). Sonic hedgehog induces angiogenesis *via* Rho kinase-dependent signalling in endothelial cells. J. Mol. Cell. Cardiol. 49, 490–498. 10.1016/j.yjmcc.2010.05.003 20478312 PMC2917529

[B131] Romagnani S. (2004). Immunologic influcents on allergy and the TH1/TH2 balance. J. Allergy Clin. Immunol. 113, 395–400. 10.1016/j.jaci.2003.11.025 14758340

[B132] Ross K. R. Darrah R. J. Hodges C. A. Lang L. Kelley T. J. (2013). Increased expression of RhoA in epithelium and smooth muscle of obese mouse models: implications for isoprenoid control of airway smooth muscle and firbroblasts. J. Allergy (Cairo) 2013, 1–11. 10.1155/2013/740973 PMC3693156 23840226

[B133] Roth M. Tamm M. (2010). The effects of omalizumab on IgE-induced asthmatic airway smooth muscle cells. Ann. Allergy Asthma Immunol. 104, 152–160. 10.1016/j.anai.2009.11.022 20306819

[B134] Roth M. Zhong J. Zumkeller C. S’ng C. T. Goulet S. Tamm M. (2013). The role of IgE-receptors in IgE-dependent airway smooth muscle cell remodelling. PLoS One 8, 1–9. 10.1371/journal.pone.0056015 PMC3573085 23457493

[B135] Saleh A. Shan L. Halayko A. J. Kung S. Gounni A. S. (2009). Critical role for STAT3 in IL-17A-mediated CCL11 expression in human airway smooth muscle cells. J. Immunol. 182, 3357–3365. 10.4049/jimmunol.0801882 19265112

[B136] Salter B. Pray C. Radford K. Martin J. G. Nair P. (2017). Regulation of human airway smooth muscle cell migration and relevance to asthma. Respir. Res. 18, 1–15. 10.1186/s12931-017-0640-8 28814293 PMC5559796

[B137] Severin F. Frezzato F. Martini V. Raggi F. Trimarco V. Martinello L. (2016). Inhibition of JAK2/STAT3 pathway leads to apoptosis in chronic lymphocytic leukemia cells. Blood 128, 20–23.

[B138] Simeone-Penney M. C. Severgnini M. Rozo L. Takahashi S. Cochran B. H. Simon A. R. (2008). PDGF-induced human airway smooth muscle cell proliferation requires STAT3 and the small GTPase Rac1. Am. J. Physiol. Lung Cell. Mol. Physiol. 294, 698–704. 10.1152/ajplung.00529.2007 18310224

[B139] Simon A. R. Takahashi S. Severgnini M. Fanburg B. L. Cochran B. H. (2002). Role of the JAK-STAT pathway in PDGF-stimulated proliferation of human airway smooth muscle cells. Am. J. Physiol. Lung Cell. Mol. Physiol. 282, 1296–1304. 10.1152/ajplung.00315.2001 12003786

[B140] Solarewicz-Madejek K. Basinski T. M. Crameri R. Akdis M. Akkaya A. Blaser K. (2009). T cells and eosinophils in bronchial smooth muscle cell death in asthma. Clin. Exp. Allergy 39, 845–855. 10.1111/j.1365-2222.2009.03244.x 19400895

[B141] Stamatiou R. Paraskeva E. Gourgoulianis K. Molyvdas P. A. Hatziefthimiou A. (2012). Cytokines and growth factors promote airway smooth muscle proliferation. ISRN Inflamm. 2012, 1–12. 10.5402/2012/731472 PMC3767366 24049651

[B142] Sui X. Kong N. Ye L. Han W. Zhou J. Zhang Q. (2014). p38 and JNK MAPK pathways control the balance of apoptosis and autophagy in response to chemotherapeutic agents. Cancer Lett. 344, 174–179. 10.1016/j.canlet.2013.11.019 24333738

[B143] Sun J. Marx S. O. Chen H. Poon M. Marks A. R. Rabbani L. E. (2001). Role for p27Kip1 in vascular smooth muscle cell migration. Circulation 103, 2967–2972. 10.1161/01.CIR.103.24.2967 11413088

[B144] Sun Y. Liu W. Liu T. Feng X. Yang N. Zhou H. (2015). Signaling pathway of MAPK/ERK in cell proliferation, differentiation, migration, senescence and apoptosis. J. Recept. Sig. Transduct. 35, 600–604. 10.3109/10799893.2015.1030412 26096166

[B145] Takeda N. Kondo M. Ito S. Ito Y. Shimokata K. Kume H. (2006). Role of RhoA inactivation in reduce cell proliferation of human airway smooth muscle by simvastatin. Am. J. Respir. Cell Mol. Biol. 35, 722–729. 10.1165/rcmb.2006-0034OC 16858009

[B146] Tamura M. Gu J. Danen E. H. J. Takino T. Miyamoto S. Yamada K. M. (1999). PTEN interactions with focal adhesion kinase and suppression of extracellular matrix-dependent phosphatidylinositol 3-kinase/Akt cell survival pathway. J. Biol. Chem. 274, 20693–20703. 10.1074/jbc.274.29.20693 10400703

[B147] Taylor D. M. Moser R. Regulier E. Breuillaud L. Dixon M. Beesen A. A. (2013). MAP kinase phosphatase 1 (MKP-1/DUSP1) is neuroprotective in Huntington’s disease *via* additive effects of JNK and p38 inhibition. J. Neurosci. 33, 2313–2325. 10.1523/JNEUROSCI.4965-11.2013 23392662 PMC3711389

[B148] Teng T. S. Lin B. Manser E. Ng D. C. H. Cao X. (2009). Stat3 promotes directional cell migration by regulating Rac1 activity *via* its activator βPIX. J. Cell Sci. 122, 4150–4159. 10.1242/jcs.057109 19861492

[B149] Tliba O. Panettieri R. A. Jr. (2009). Noncontractile function of airway smooth muscle cells in asthma. Annu. Rev. Physiol. 71, 509–535. 10.1146/annurev.physiol.010908.163227 18851708

[B150] Vignola A. M. Chanez P. Chaippara G. Merendino A. Pace E. Rizzo A. (1997). Transforming growth factor-β expression in mucosal biopsies in asthma and chronic bronchitis. Am. J. Respir. Crit. Care Med. 156, 591–599. 10.1164/ajrccm.156.2.9609066 9279245

[B151] Walker T. R. Moore S. M. Lawson M. F. Panettieri R. A. Jr. Chilvers E. R. (1998). Platelet-derived growth factor-BB and thrombin activate phosphoinositide 3-kinase and protein kinase B: role in mediating airway smooth muscle proliferation. Mol. Pharmacol. 54, 1007–1015. 10.1124/mol.54.6.1007 9855629

[B152] Wang H. Yao H. Yi B. Kazama K. Liu Y. Deshpande D. (2018a). MicroRNA-638 inhibits human airway smooth muscle cell proliferation and migration through targeting cyclin D1 and NOR1. J. Cell. Physiol. 231, 1–13. 10.1002/jcp.26930 PMC6202131 30076719

[B153] Wang J. Wang H. S. Su Z. B. (2018b). MicroRNA-142 inhibits proliferation and promotes apoptosis in airway smooth muscle cells during airway remodeling in asthmatic rats *via the* inhibition of TGF-β-dependent EGFR signalling pathway. Cell. Physiol. Biochem. 47, 1682–1695. 10.1159/000490986 29949788

[B154] Wang Q. Li H. Yao Y. Lu G. Wang Y. Xia D. (2016). HB-EGF-promoted airway smooth muscle cells and their progenitor migration contribute to airway smooth muscle remodeling in asthmatic mouse. J. Immunol. 196, 2361–2367. 10.4049/jimmunol.1402126 26826248

[B155] Wei L. Liu Y. Kaneto H. Fanburg B. L. (2010). JNK regulates serotonin-mediated proliferation and migration of pulmonary artery smooth muscle cells. Am. J. Physiol. Lung Cell. Mol. Physiol. 298, 863–869. 10.1152/ajplung.00281.2009 PMC2886609 20228179

[B156] Will M. Qin A. C. Toy W. Yao Z. Rodrik-Outmezguine V. Schneider C. (2014). Rapid induction of apoptosis by PI3K inhibitors is dependent upon their transient inhibition of RAS-ERK signaling. Cancer Discov. 4, 334–347. 10.1158/2159-8290.CD-13-0611 24436048 PMC4049524

[B157] Woodruff P. G. Dolganov G. M. Ferrando R. E. Donnelly S. Hays S. R. Solberg O. D. (2004). Hyperplasia of smooth muscle in mild to moderate asthma without changes in cell size or gene expression. Am. J. Respir. Crit. Care Med. 169, 1001–1006. 10.1164/rccm.200311-1529OC 14726423

[B158] Xia Y. C. Redhu N. S. Moir L. M. Koziol-White C. Ammit A. J. Al-Alwan L. (2013). Pro-inflammatory and immunomodulatory functions of airway smooth muscle: emerging concepts. Pulm. Pharmacol. Ther. 26, 64–74. 10.1016/j.pupt.2012.05.006 22634303

[B159] Yan Y. Luo Y. Zhong M. Shao L. (2018). MiR-216a inhibits proliferation and promotes apoptosis of human airway smooth muscle cells by targeting JAK2. J. Asthma 9, 1–9. 10.1080/02770903.2018.1509991 30299194

[B160] Yang G. Volk A. Petley T. Emmell E. Giles-Komar J. Shang X. (2004). Anti-IL-13 monoclonal antibody inhibits airway hyperresponsiveness, inflammation and airway remodeling. Cytokine 28, 224–232. 10.1016/j.cyto.2004.08.007 15566951

[B161] Yao Q. Renault M. Chapouly C. Vandierdonck S. Belloc I. Jaspard-Vinassa B. (2014). Sonic hedgehog mediates a novel pathway of PDGF-BB-dependent vessel maturation. Blood 123, 2429–2437. 10.1182/blood-2013-06-508689 24472833

[B162] Ye Q. He X. D’Urzo A. (2017). A review on the safety and efficacy of inhaled corticosteroids in the management of asthma. Pulm. Ther. 3, 1–18. 10.1007/s41030-017-0043-5

[B163] Ye X. Hong W. Hao B. Peng G. Huang L. Zhao Z. (2018). PM2.5 promotes human bronchial smooth muscle cell migration *via* the sonic hedgehog signaling pathway. Respir. Res. 19, 1–11. 10.1186/s12931-017-0702-y 29499705 PMC5833105

[B164] Zeke A. Misheva M. Remenyi A. Bogoyevitch M. A. (2016). JNK signaling: regulation and functions based on complex protein–protein partnerships. Microbiol. Mol. Biol. Rev. 80, 793–835. 10.1128/MMBR.00043-14 27466283 PMC4981676

[B165] Zhang H. Sun Z. Yu L. Sun J. (2017a). Mir-139-5p inhibits proliferation and promoted apoptosis of human airway smooth muscle cells by downregulating the Brg1 gene. Respir. Physiol. Neurobiol. 246, 9–16. 10.1016/j.resp.2017.07.004 28711603

[B166] Zhang T. Liao J. Yu L. Liu G. (2017b). Regulating effect of glycyrrhetinic acid on bronchial asthma smooth muscle proliferation and apoptosis as well as inflammatory factor expression through ERK1/2 signaling pathway. Asian Pac. J. Trop. Med. 10, 1172–1176. 10.1016/j.apjtm.2017.10.025 29268974

[B167] Zhang Y. Xue Y. Liu Y. Song G. Lv G. Wang Y. (2016a). MicroRNA-146a expression inhibits the proliferation and promotes the apoptosis of bronchial smooth muscle cells in asthma by directly targeting the epidermal growth factor receptor. Exp. Ther. Med. 12, 854–858. 10.3892/etm.2016.3427 27446287 PMC4950248

[B168] Zhang Z. Yu J. Yang T. Xu D. Chi D. Xia Y. (2016b). A novel c-Jun N-terminal kinase (JNK) signaling complex involved in neuronal migration during brain development. J. Biol. Chem. 291, 11466–11475. 10.1074/jbc.M116.716811 27026702 PMC4882418

[B169] Zhao L. Kuang H. Zhang L. Wu J. Chen X. Zhang X. (2014). Effect of TRPV1 channel on proliferation and apoptosis of airway smooth muscle cells of Rats. J. Huazhong Univ. Sci. Technol. Med. Sci. 34, 504–509. 10.1007/s11596-014-1306-0 25135718

[B170] Zhao X. Guan J. (2011). Focal adhesion kinase and its signaling pathways in cell migration and angiogenesis. Adv. Drug Deliv. Rev. 63, 610–615. 10.1016/j.addr.2010.11.001 21118706 PMC3132829

